# Self‐Boosting Programmable Release of Multiple Therapeutic Agents by Activatable Heterodimeric Prodrug‐Enzyme Assembly for Antitumor Therapy

**DOI:** 10.1002/advs.202409960

**Published:** 2024-11-21

**Authors:** Shanshan Jiang, Bhaskar Gurram, Junfei Zhu, Shan Lei, Yifan Zhang, Ting He, Oya Tagit, Hui Fang, Peng Huang, Jing Lin

**Affiliations:** ^1^ Marshall Laboratory of Biomedical Engineering International Cancer Center, Guangdong Key Laboratory for Biomedical Measurements and Ultrasound Imaging, Laboratory of Evolutionary Theranostics School of Biomedical Engineering Shenzhen University Medical School Shenzhen University Shenzhen 518055 China; ^2^ Nanophotonics Research Center Shenzhen Key Laboratory of Micro‐Scale Optical Information Technology Institute of Microscale Optoelectronics Shenzhen University Shenzhen 518060 China; ^3^ Department of BioInterfaces Institute for Chemistry and Bioanalytics School of Life Sciences FHNW University of Applied Sciences and Arts Northwestern Switzerland Muttenz 4132 Switzerland

**Keywords:** antitumor therapy, glucose oxidase, heterodimeric prodrug, reactive oxygen species, self‐boosted programmable release

## Abstract

Endogenous stimuli‐responsive prodrugs, due to their disease lesion specificity and reduced systemic toxicity, have been widely explored for antitumor therapy. However, reactive oxygen species (ROS) as classical endogenous stimuli in the tumor microenvironment (TME) are not enough to achieve the expected drug release. Herein, a ROS‐activatable heterodimeric prodrug‐loaded enzyme assembly is developed for self‐boosting programmable release of multiple therapeutic agents. The heterodimeric prodrug NBS‐TK‐PTX (namely NTP) is composed of 5‐(ethylamino)‐9‐diethylaminobenzo[*a*]phenothiazinium chloride analog (NBS), paclitaxel (PTX) and ROS‐responsive thioketal (TK) linker, which shows a strong binding affinity with glucose oxidase (GOx), thus obtaining NTP@GOx assembly. Notably, the enzymatic activity of GOx in NTP@GOx is inhibited by NTP. The programmable release is achieved by following steps: i) NTP@GOx is partially dissociated in acidic TME, thus releasing a small segment of NTP and GOx. Thereupon, the enzymatic activity of GOx is recovered; ii) GOx‐triggered pH reduction further facilitates the dissociation of NTP@GOx, thus accelerating a large amount of NTP and GOx release; iii) The TK linker of prodrug NTP is cleaved by hydrogen peroxide generated by GOx catalysis, thus expediting the release of NBS for Type‐I photodynamic therapy and PTX for chemotherapy, respectively. The NTP@GOx shows great potential for multimodal synergistic cancer therapy.

## Graphical abstract

An activatable heterodimeric prodrug‐glucose oxidase assembly (NTP@GOx) is developed for fluorescence (FL) and photoacoustic (PA) dual‐modality imaging navigated glucose oxidase (GOx)‐mediated starvation therapy, paclitaxel (PTX)‐induced chemotherapy and photosensitizer (NBS)‐instructed Type‐I photodynamic therapy (PDT) of tumors.

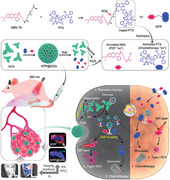

## 1 Introduction

Chemotherapy is a traditional cancer treatment.^[^ ^1^, ^2^ ^]^ However, chemotherapeutic drugs usually have highly toxic side effects due to their inability to distinguish between normal and tumor cells.^[^ ^3^, ^4^, ^5^ ^]^ By taking the advantage of irregular metabolism of tumors,^[^ ^6^, ^7^, ^8^ ^]^ tumor microenvironment (TME)‐responsive prodrugs have been explored based on endogenous stimuli, such as hypoxia, overexpressed enzymes, acidity, reactive oxygen species (ROS), and so on.^[^ ^9^, ^10^, ^11^, ^12^, ^13^, ^14^, ^15^, ^16^ ^]^ Among them, ROS as key metabolites have attracted great interest. So far, a series of ROS‐sensitive groups (e.g., propylene sulfide,^[^ ^17^ ^]^ boronic acid esters,^[^ ^18^, ^19^, ^20^ ^]^ thioketones,^[^ ^21^, ^22^, ^23^ ^]^ tellurium,^[^ ^24^, ^25^ ^]^ selenium,^[^ ^26^ ^]^ ferrocene,^[^ ^27^, ^28^, ^29^ ^]^ and anthocyanins^[^ ^30^ ^]^) have been developed to construct ROS‐activatable prodrugs. However, the content of ROS in TME is not enough to achieve the expected drug release.^[^ ^31^ ^]^ Therefore, the development of ROS‐activatable prodrugs with self‐boosting properties is a big challenge.

Glucose oxidase (GOx)‐induced starvation therapy shows great potential in cancer treatment, wherein GOx specifically catalyzes *β*‐D‐glucose oxidation to generate large amounts of hydrogen peroxide (H_2_O_2_).^[^ ^32^, ^33^ ^]^ The increased H_2_O_2_ level is likely to enhance the activation efficiency of ROS‐activatable prodrugs.^[^ ^34^ ^]^ However, the oxygen (O_2_) was consumed during the oxidation, causing tumor hypoxia severely, which weakens the efficacy of O_2_‐dependent antitumor drugs, such as Type‐II photosensitizers (PSs).^[^ ^35^, ^36^, ^37^, ^38^, ^39^ ^]^ By contrast, Type‐I PSs are good candidates due to their low O_2_‐dependency.^[^ ^40^ ^]^ For example, 5‐(ethylamino)‐9‐diethylaminobenzo[*a*]phenothiazinium chloride analog (NBS) is used as Type‐I PS for photodynamic therapy (PDT) to generate superoxide radical (O_2_^−•^) via Haber Weiss/Fenton reaction under 660 nm laser irradiation, even at a low O_2_ level.^[^ ^41^ ^]^ Therefore, the combination of GOx with O_2_‐independent ROS‐activatable prodrugs could enhance drug release and achieve effective synergy between starvation and other therapies.

**Scheme 1:**
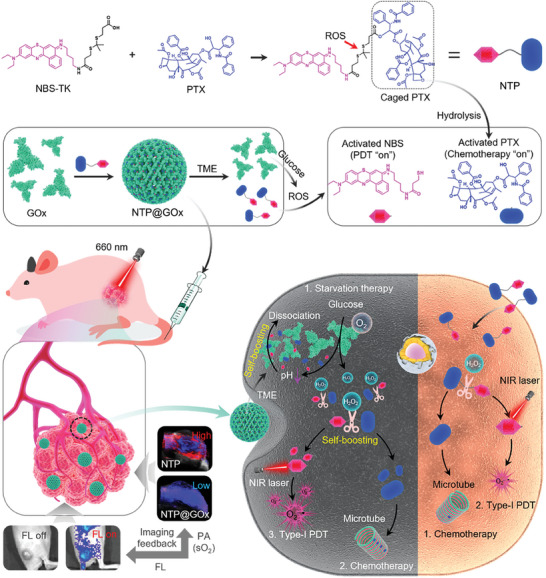
Schematic illustration of ROS‐activatable heterodimeric prodrug‐loaded enzyme assembly (NTP@GOx) for self‐boosting programmable release of multiple therapeutic agents against tumors.

Herein, we developed a ROS‐activatable heterodimeric prodrug‐loaded enzyme assembly for self‐boosting programmable release of multiple therapeutic agents (**Scheme** **1**). The heterodimeric prodrug NBS‐TK‐PTX (namely NTP) was composed of NBS, paclitaxel (PTX), and ROS‐responsive thioketal (TK) linker. The NTP showed a strong binding affinity with GOx (−8.1 kcal mol^−1^) due to the hydrophobic interaction, thus obtaining NTP@GOx assembly. Notably, the enzymatic activity of GOx in NTP@GOx was inhibited by NTP. The programmable release was achieved by following steps: i) NTP@GOx was partially dissociated in acidic TME, thus releasing a small segment of NTP and GOx. Thereupon, the enzymatic activity of GOx was recovered; ii) GOx‐triggered pH reduction further facilitated the dissociation of NTP@GOx, thus accelerating a large amount of NTP and GOx release; iii) The TK linker of prodrug NTP was cleaved by H_2_O_2_ generated by GOx catalysis, thus expediting the release of NBS for Type‐I PDT and PTX for chemotherapy, respectively. Under the navigation of fluorescence (FL) and photoacoustic (PA) dual‐modality imaging, NTP@GOx exhibited the synergistic therapeutic effect of GOx‐mediated starvation therapy, PTX‐induced chemotherapy, and NBS‐based Type‐I PDT.

## 2 Results and Discussion

### 2.1 Synthesis and Characterization of NTP@GOx

**Figure 1:**
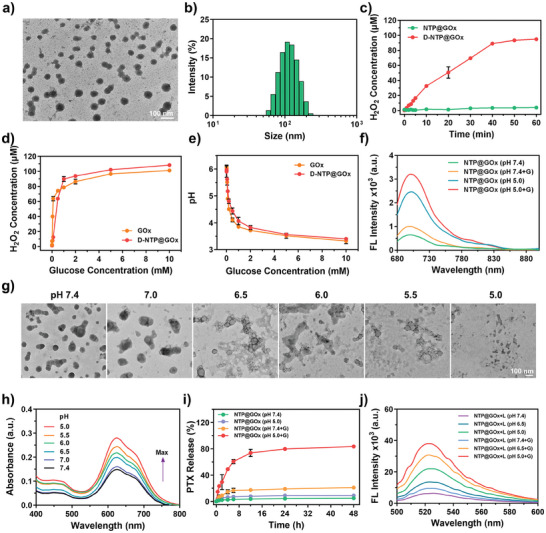
Characterizations of NTP@GOx. a) TEM image and b) Size distribution of NTP@GOx. Scale bar: 100 nm. c) H_2_O_2_ generation of NTP@GOx and dissociated NTP@GOx (denoted as D‐NTP@GOx) incubated with glucose at various time. d) H_2_O_2_ generation and e) pH value of GOx and D‐NTP@GOx after 1 h incubation with various concentrations of glucose. f) The FL spectra of NTP@GOx with different treatments. g) TEM images of NTP@GOx after incubation in PBS with different pH values. Scale bar: 100 nm. h) NTP release behaviors of NTP@GOx in PBS with different pH values. i) PTX release behaviors of NTP@GOx in different solutions. j) The FL spectra of DHR123 with different treatments. G: 10 mM glucose, L: 660 nm laser irradiation. Data are means ± SD, *n* = 3.

The NTP was synthesized by the conjugation of NBS and PTX with a ROS‐sensitive TK linker (Figure S1, Supporting Information). The structure was confirmed by high‐resolution mass spectra (HR‐MS) and nuclear magnetic resonance spectra (^1^H‐NMR and ^13^C‐NMR) (Figures S2–S7, Supporting Information). The H_2_O_2_ response of NTP to release NBS and PTX was investigated. The peak areas of NBS and PTX in high‐performance liquid chromatography (HPLC) increased with the addition of H_2_O_2_, indicating that NBS and PTX can be released from NTP under H_2_O_2_ stimulation (Figure S8, Supporting Information). Next, GOx and NTP were assembled through hydrophobic interaction.^[^ ^42^, ^43^ ^]^ Specifically, the disulfide bond of GOx was cleaved by the *β*‐mercaptoethanol, and then assembled with NTP, thus obtaining the final product NTP@GOx. A molecular docking simulation was performed to investigate the interaction between GOx and NTP. The binding affinity of NTP with GOx was stronger (−8.1 kcal mol^−1^) than that of NBS (−6.3 kcal mol^−1^) (Figure S9, Supporting Information). The transmission electron microscope (TEM) image confirmed the spherical morphology of the as‐prepared NTP@GOx (**Figure** **1**a). The average hydrodynamic diameter of NTP@GOx in phosphate buffer solution (PBS, pH 7.4) was 137.5 nm with a polydispersity index (PDI) of 0.135 (Figure 1b). Moreover, the size and PDI of NTP@GOx showed no obvious change within 7 days of storage in different media (Figure S10, Supporting Information), demonstrating its good stability in physiological conditions. The zeta potential of NTP@GOx (−2.61 ± 0.32 mV) was neutralized compared to that of GOx (−7.94 ± 0.34 mV), indicating the successful loading of NTP (Figure S11, Supporting Information). Additionally, NTP@GOx exhibited characteristic absorption peaks of both NTP and GOx (Figure S12a, Supporting Information), and presented protein bands of GOx in sodium dodecyl sulfate‐polyacrylamide gel electrophoresis (SDS‐PAGE) diagram (Figure S12b, Supporting Information). These results verified the presence of both NTP and GOx in NTP@GOx, confirming its successful assembling. The maximum encapsulation efficiency and loading efficiency of NTP in NTP@GOx were calculated to be 57.4% and 4.4%, respectively, when the mass ratio of NTP to GOx was 2.5% (Figure S13, Supporting Information). Besides, the FL signal of NTP in NTP@GOx was quenched relative to the free NTP (Figure S14, Supporting Information).

Next, the enzymatic activity of NTP@GOx was evaluated. As shown in Figure 1c, the generation of H_2_O_2_ (4.16 µM) in the solution of NTP@GOx and glucose was quite low. Notably, the generation of H_2_O_2_ (95.1 µM) in the solution of NTP@GOx pretreated with acidic PBS buffer (pH 5.0) (denoted as D‐NTP@GOx) was dramatically increased, indicating that the enzymatic activity of GOx was significantly inhibited in NTP@GOx but recovered under acidic conditions. These results are likely attributed to secondary structure changes of GOx under acidic conditions, which affect its ability to bind to small molecules, subsequently leading to the degradation of the assembly and the release of active GOx.^[^ ^44^ ^]^ The enzymatic activity of D‐NTP@GOx was further compared with free GOx. In the presence of glucose, the changes in H_2_O_2_ concentration (Figure 1d) and pH values (Figure 1e) with the concentration of glucose in D‐NTP@GOx were similar to that of free GOx, which was consistent with the previous studies.^[^ ^44^ ^]^ Furthermore, the Michaelis‐Menten constant (*K*_m_) and maximum velocity (*V*_max_) of D‐NTP@GOx were calculated to be 53.49 mM and 1.0890 mM min^−1^, respectively, which are comparable to those of GOx and significantly higher than those of GOx@NTP (Figure S15 and Table S1, Supporting Information). We assumed that the recovery of enzymatic activity is attributed to the acid‐responsive dissociation of NTP@GOx. As shown in Figure 1f, the FL intensity of NTP@GOx was gradually recovered as pH decreased and further enhanced in the presence of glucose, which should be attributed to the NTP release during NTP@GOx dissociation process. The TEM images showed the NTP@GOx remained intact morphology at pH 7.4, while completely dissociated into small particles at pH 5.0 (Figure 1g), which further verified the acid‐responsive dissociation of NTP@GOx. Afterward, the acid‐dependent release of NTP from NTP@GOx was evaluated under different pH conditions. We first investigated the absorbance changes of free NTP in different pH solutions including ethanol (Figure S16a, Supporting Information) and PBS (Figure S16b, Supporting Information). The results verified NTP could maintain good stability in different pH solutions. Interestingly, when NTP@GOx was treated with different pH solutions, the absorption intensities of NTP increased with pH decrease, indicating an acid‐enhanced NTP release from NTP@GOx (Figure 1h). Next, the ROS‐responsive release of PTX and NBS from NTP was investigated in the presence of glucose. As shown in Figure 1i, the release rate of PTX in NTP@GOx was 83.57% at pH 5.0 with 10 mM of glucose, which was much higher than that without glucose (8.98%), and that at pH 7.4 with or without glucose (21.03% and 4.81%, respectively). Similarly, the coexistence of acid and glucose also accelerated the release of NBS from NTP@GOx (Figure S17, Supporting Information). The abundant release of PTX and NBS was attributed to the generation of H_2_O_2_ by GOx catalysis, which can cleave the TK linker. Subsequently, electron spin resonance (ESR) analysis was conducted to determine the types of ROS generated by NTP@GOx upon irradiation. The results confirmed that O_2_^−•^ is the primary type (Figure S18, Supporting Information). After that, dihydroerhodamine 123 (DHR123) was employed to assess the O_2_^−•^ generation efficiency of NTP@GOx (Figure 1j; Figure S19, Supporting Information). FL signal of DHR123 at 525 nm (FL_525_) in NTP@GOx plus laser treated group (NTP@GOx+L) exhibited an acidity‐dependent manner no matter with or without glucose, further indicating the acidic‐enhanced release of NTP which generated robust O_2_^−•^. Noteworthily, at pH 5.0, the FL_525_ intensity of DHR123 in NTP@GOx plus glucose and laser irradiation group (NTP@GOx+G+L) displayed a 5.14‐fold enhancement compared with that of non‐glucose treated group, indicating glucose‐accelerated O_2_^−•^ generation. Furthermore, the FL_525_ intensity of DHR123 dramatically decreased with the addition of O_2_^−•^ scavenger (Vitamin‐c, Vc).^[^ ^20^ ^]^ The above results verified that NTP@GOx in an acid and glucose‐rich TME under laser irradiation can generate O_2_^−•^ for Type‐I PDT.

### 2.2 In Vitro ROS Generation and Intracellular Accumulation Evaluation

**Figure 2:**
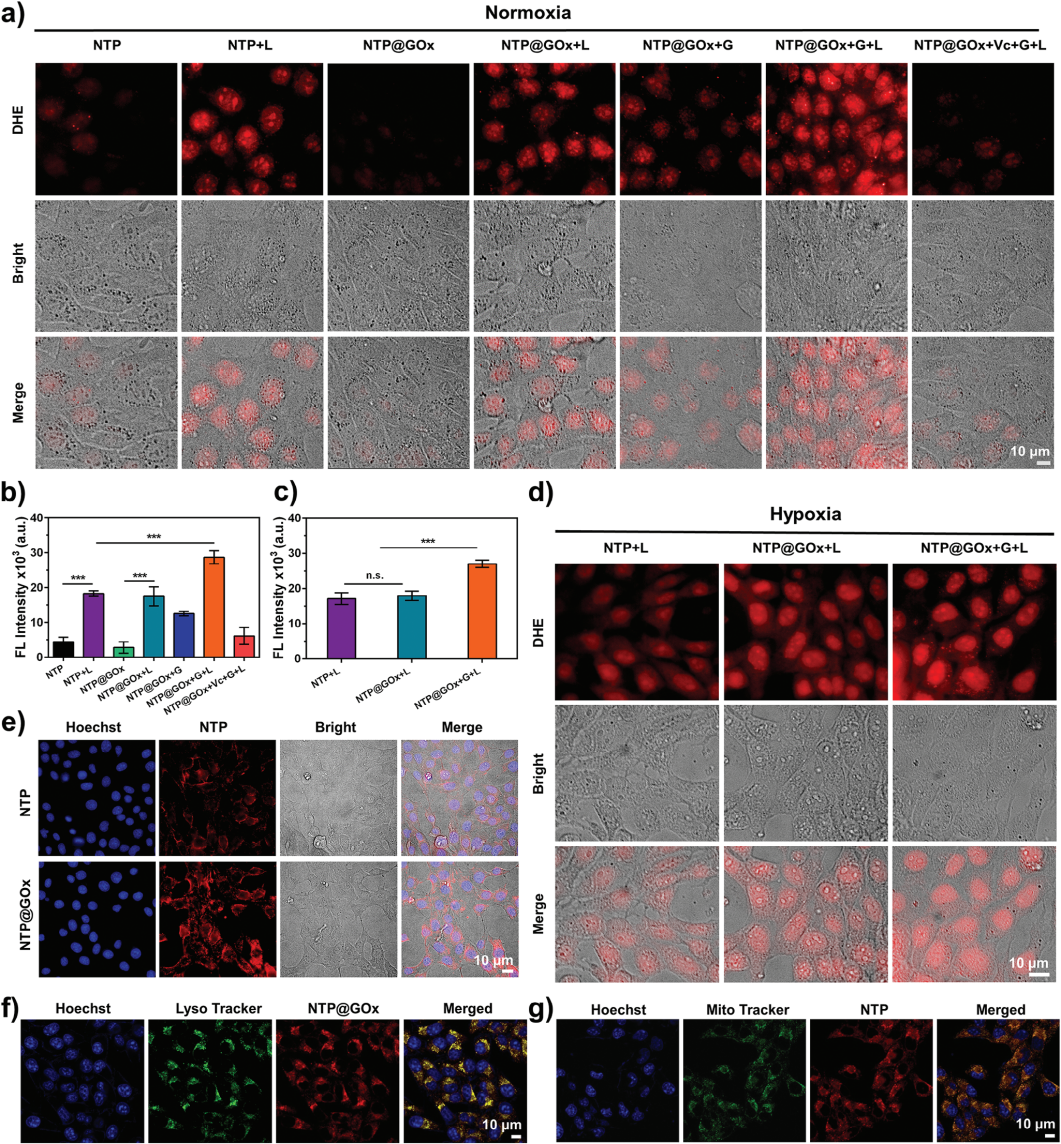
In vitro ROS generation and intracellular accumulation evaluation. a) DHE‐stained FL images and b) quantification of FL intensity of cells after different treatments under normoxia. G: 10 mm glucose, L: 660 nm laser irradiation. Scale bar: 10 µM. c) FL intensity and d) images of DHE in 4T1 cells after different treatments under hypoxia. G: 10 mM glucose, L: 660 nm laser irradiation. Scale bar: 10 µM. e) FL images of Hoechst (blue, 10 µM) and NTP/NTP@GOx (red) co‐stained 4T1 cells after incubation with NTP@GOx or NTP. Scale bar: 10 µM. f) FL images of Hoechst (blue, 10 µM), Lyso Tracker (green, 100 nM), and NTP@GOx (red) co‐stained 4T1 cells. Scale bar: 10 µM. g) FL images of Hoechst (blue, 10 µM), Mito Tracker (green, 100 nM), and NTP@GOx (red) co‐stained 4T1 cells. Scale bar: 10 µM. In Figure 2a–g, NTP@GOx: 1.15 µg mL^−1^; NTP: 50 ng mL^−1^. Data are means ± SD, *n* = 4–6.

The intracellular levels of ROS were detected using a 2′,7′‐dichlorodihydrofluorescein diacetate (DCFH‐DA) probe. In the absence of glucose, no significant change in the FL intensity of DCFH‐DA was observed with the increase in the concentration of NTP@GOx (Figure S20a, Supporting Information). Interestingly, the FL intensity showed a concentration‐dependent increase upon co‐incubation with NTP@GOx and glucose (Figure S20b, Supporting Information), which indicated the enhanced intracellular ROS generation with glucose addition, consequently accelerating the cleavage of TK linker in NTP. Afterward, in vitro O_2_^−•^ generation by NTP@GOx under normoxia and hypoxia was investigated on 4T1 murine breast cancer cells (4T1 cells). In normoxia, NTP or NTP@GOx treated cells exhibited low FL intensity of dihydroethidium (DHE, a O_2_^−•^ probe) no matter with or without glucose (**Figure** **2**a). Interestingly, upon laser irradiation, the NTP@GOx and NTP‐treated groups without glucose exhibited 6.19‐ and 4.12‐fold enhancements in FL intensity of DHE, indicating the laser‐triggered O_2_^−•^ generation (Figure 2a,b). Noticeably, the FL signal of the NTP@GOx+G+L group was 1.64‐fold enhanced compared with that of the NTP@GOx+L group, indicating more amount of NBS released from NTP@GOx with glucose addition and causing more O_2_^−•^ generated. These results were attributed to GOx catalysis‐induced H_2_O_2_ generation which promotes NBS release. The enhanced FL intensity of DHE could be significantly reduced by the addition of Vc, which is consistent with the DHR123 staining results. Similarly, almost no intracellular FL signal of DHE was observed in NTP or NTP@GOx‐treated 4T1 cells under hypoxia (Figure S21, Supporting Information). The FL intensity of DHE was significantly increased under laser irradiation, which was further enhanced by glucose addition (Figure 2c,d). These results indicated that the NTP@GOx could perform O_2_‐independent Type‐I PDT in 4T1 cells.

Then, in vitro cellular uptake of NTP@GOx in 4T1 cells was determined. After 8 h of incubation with 4T1 cells, NTP@GOx exhibited strong FL intensity (1.7‐fold) in cells compared to free NTP (Figure 2e; Figure S22, Supporting Information). Furthermore, the real‐time cellular uptake and metabolic profiles showed that NTP and NTP@GOx reached maximum intracellular uptake after 6 and 8 h of incubation, respectively, and gradually decreased over time (Figure S23, Supporting Information). Notably, compared with the free NTP, the NTP@GOx group showed a stronger FL signal at each time point after 8 h of incubation. The above results suggested that the uptake of NTP@GOx was more efficient in 4T1 cells and the retention rate was higher in cells than that of the free NTP. Additionally, a weaker FL signal of NTP@GOx was observed in normal cells (human embryonic kidney cells, HEK293T cells) compared to tumor cells, due to the more neutral pH and lower glucose level in normal cells, which prevented degradation of NTP@GOx, thus reducing the release of NTP (Figure S24a,b, Supporting Information). Subsequently, we assessed the localization of NTP@GOx in subcellular organelles of 4T1 cells. Most of NTP@GOx was localized in lysosomes by the endocytosis with a Pearson correlation coefficient of 0.93 (Figure 2f). Additionally, the red FL signal was also observed in mitochondria with a Pearson correlation coefficient of 0.87 (Figure 2g). The observations demonstrated that after entering lysosomes, NTP@GOx was degraded under acidic conditions, then the NTP was released and targeted to mitochondria.

### 2.3 In Vitro Cytotoxicity Evaluation

**Figure 3:**
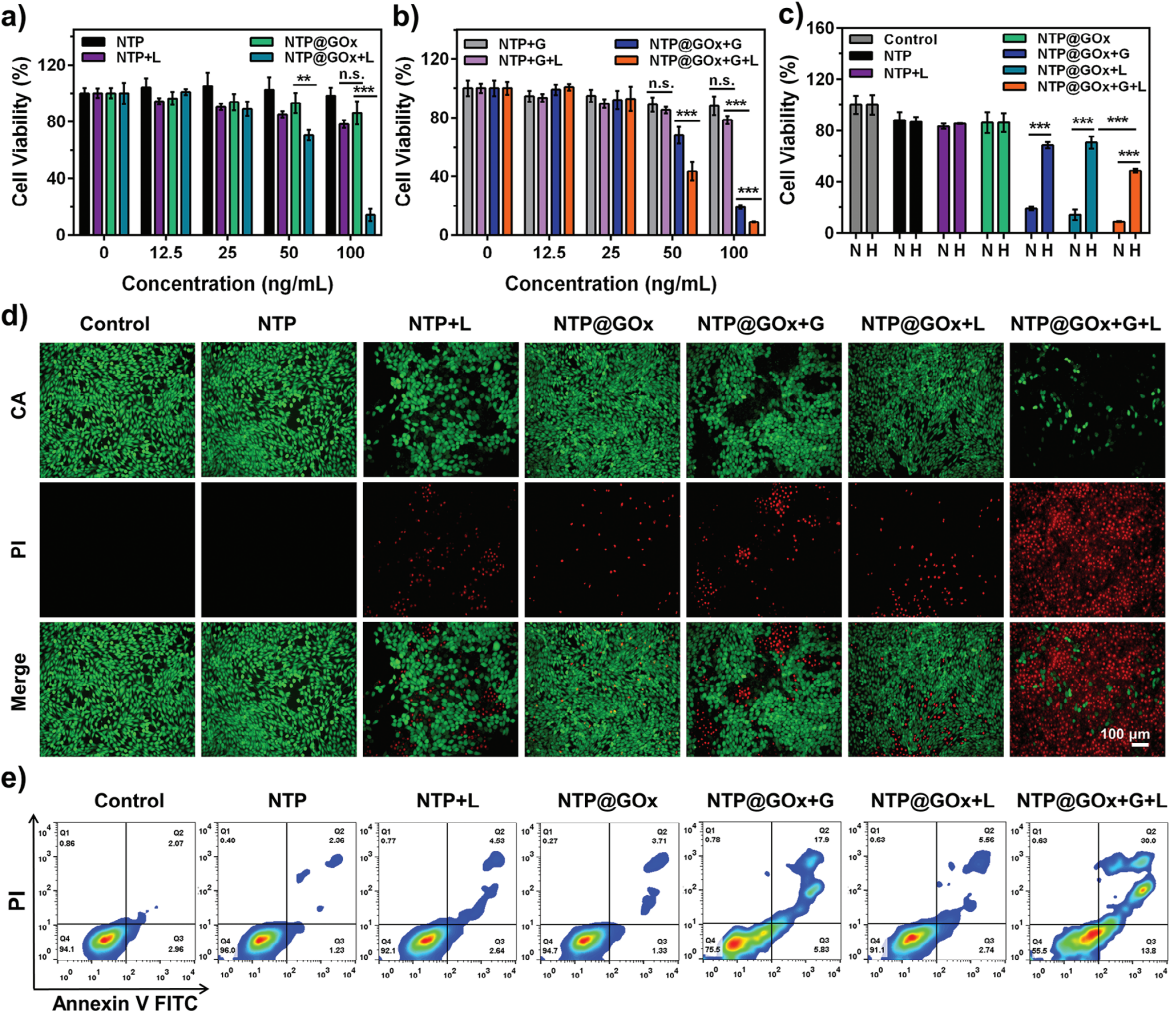
In vitro cytotoxicity evaluation. Relative viabilities of 4T1 cells in a) glucose‐free and b) glucose‐containing conditions with different treatments. c) Cytotoxicity of different treatments under normoxia (labeled as N) and hypoxia (labeled as H). d) Calcein AM (CA) and Propidium Iodide (PI) co‐staining images of 4T1 cells with various treatments. Scale bar: 100 µM. e) Flow cytometric analysis of apoptosis of 4T1 cells after different treatments for 24 h. G: glucose, L: 660 nm laser irradiation. Data are means ± SD, *n* = 3.

The cytotoxicity of NTP@GOx was evaluated under normoxic and hypoxic conditions. We initially investigated the toxicity of NTP@GOx on HEK293T cells. As a result, the NTP@GOx showed a survival rate of cells over 95% even at a high concentration (400 ng mL^−1^), indicating its good biocompatibility (Figure S24c, Supporting Information). In dark conditions, both NTP and NTP@GOx showed negligible cytotoxicity in a glucose‐free cell medium (**Figure** **3**a). At the NTP concentration of 50 ng mL^−1^, NTP@GOx induced 4T1 cellular inhibition rate was 56.75% after laser irradiation in a glucose‐containing cell medium (Figure 3b), which was higher than that of laser irradiation alone (29.32%) (Figure 3a) or glucose alone (31.81%) (Figure 3b). Besides, for the NTP@GOx+G+L group, the half maximal inhibitory concentration (IC_50_) for 4T1 cells was 47 ng mL^−1^, which was significantly lower than that of other treatment groups (Table S2, Supporting Information). Hypoxia limited the catalytic efficiency of GOx, resulting in a lower tumor‐killing efficiency than normoxia.^[^ ^32^, ^33^ ^]^ Particularly, the NTP@GOx plus laser irradiation caused tumor cell death via Type‐I PDT under hypoxia, which was further enhanced after glucose addition (Figure 3c). The results of live/dead assays and flow cytometry further verified the good therapeutic efficacy of NTP@GOx plus laser irradiation, especially in the presence of glucose (Figure 3d,e). These results suggested that the NTP@GOx could perform effective starvation/Type‐I PDT/chemotherapy in vitro.

### 2.4 In Vivo FL/PA Imaging Assessment

**Figure 4:**
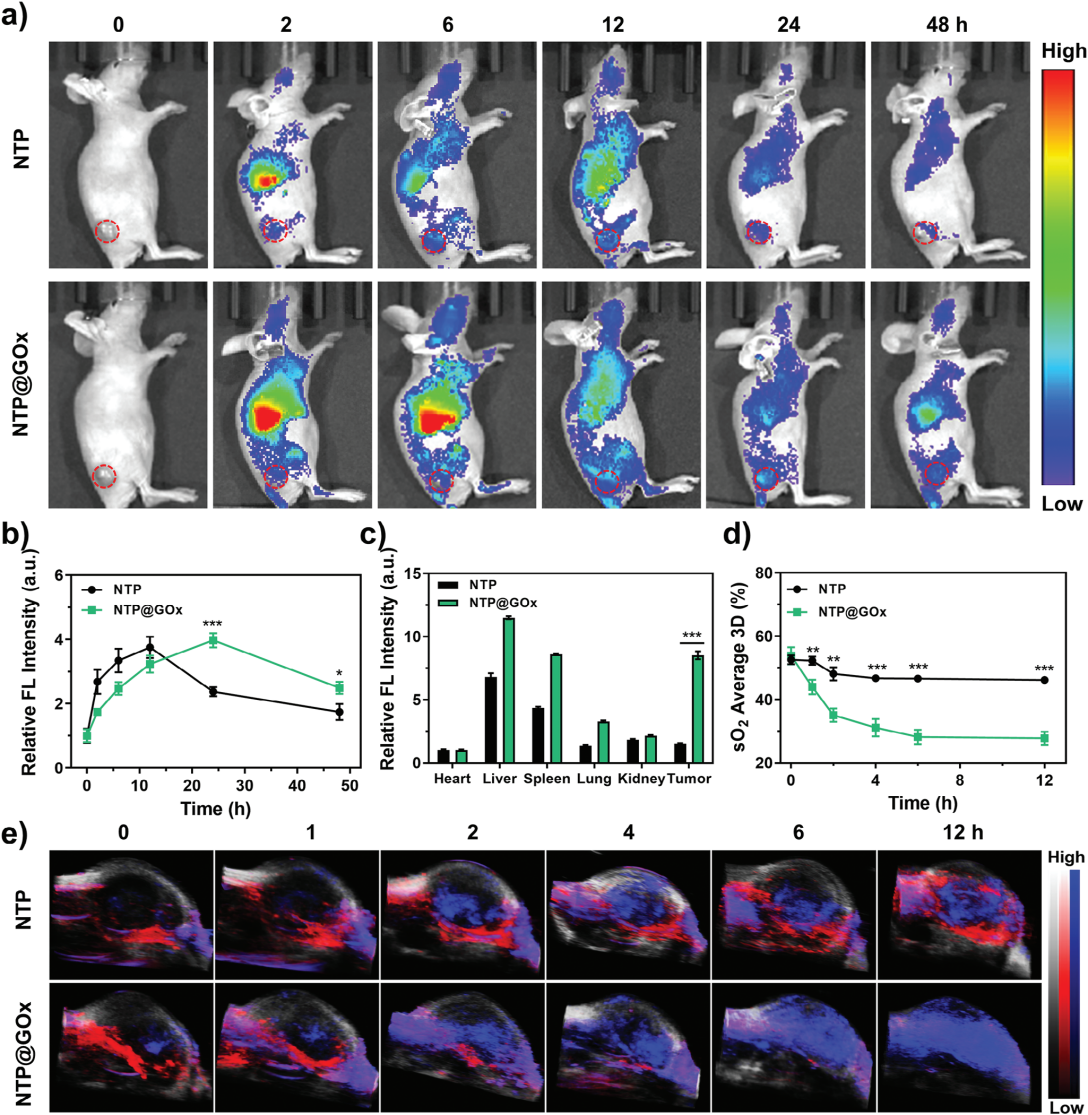
In vivo FL/PA imaging assessment. a) FL images and b) relative FL intensity of 4T1 tumor‐bearing mice after i.v. injection of NTP@GOx or NTP, respectively. c) Relative FL intensity of tumors and major organs at 24 h post‐injection. d) O_2_ average percentage and e) PA images of O_2_ levels in tumor regions after indicated treatments. Grey: B‐mode; Red: OxyHb; Blue: DeoxyHb. Data are means ± SD, *n* = 3.

The biocompatibility and tumor accumulation properties of NTP@GOx were evaluated. Even at a high concentration (200 µg mL^−1^), the hemolysis rate of NTP@GOx was less than 2.5% (Figure S25, Supporting Information), further indicating that NTP@GOx has good blood biocompatibility. Next, in vivo tumor accumulation of NTP@GOx was investigated on 4T1 tumor‐bearing nude mice by FL imaging. The FL intensity of the tumor area increased over time and peaked at 24 h after intravenous (i.v.) injection of NTP@GOx. Besides, NTP@GOx has a higher tumor accumulation and longer retention compared to NTP (**Figure** **4**a,b), which facilitated stronger PDT efficacy. Meanwhile, the FL images of the ex vivo tumor showed a 5.2‐fold enhancement in intensity for the group of NTP@GOx compared to that for the group of NTP (Figure 4c; Figure S26, Supporting Information). Given the O_2_‐consuming catalytic capacity of GOx, the intratumoral O_2_ level was assessed by PA imaging in vivo. NTP@GOx‐treated tumor‐bearing mice showed a strong deoxyhemoglobin (DeoxyHb) signal (Figure 4d,e), indicating elevated levels of tumor hypoxia during treatment. However, the degree of intratumoral hypoxia was not significantly affected by NTP. The observations indicated that NTP@GOx could cause tumor hypoxia in starvation therapy and also demonstrated the feasibility of combining GOx‐mediated starvation therapy with NTP‐triggered Type‐I PDT.

### 2.5 In Vivo Antitumor Efficiency Evaluation

**Figure 5:**
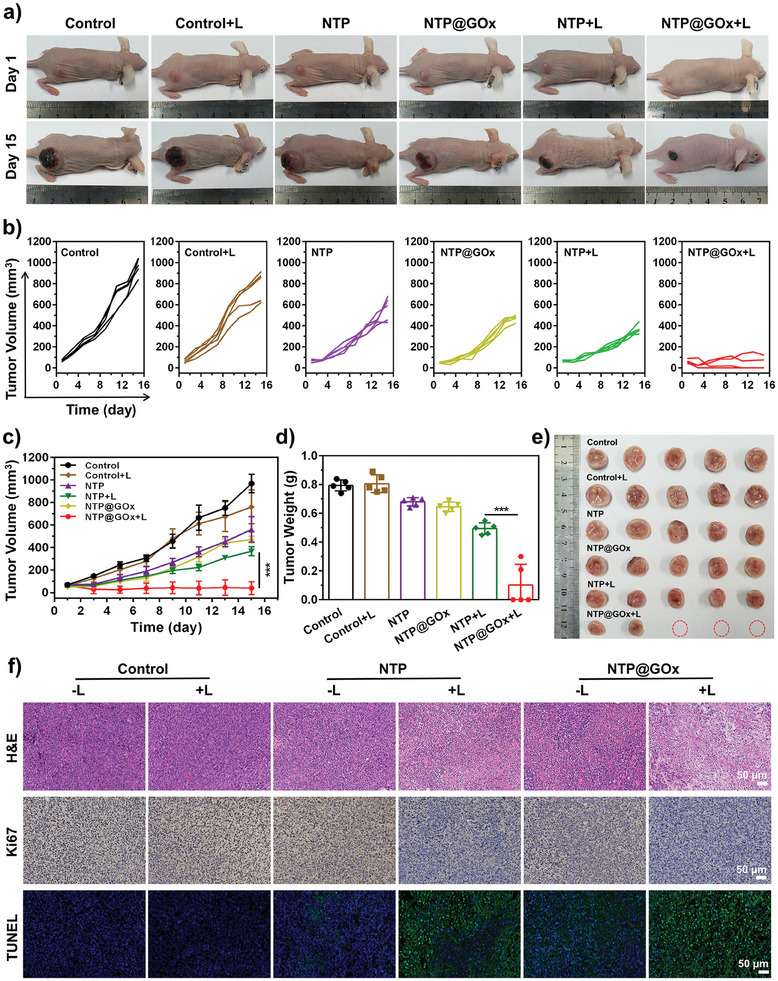
In vivo antitumor efficiency evaluation. a) Digital photographs of mice on the 1st day and the 15th day after indicated treatments. b) Tumor growth curves, c) tumor volume, and d) tumor weight of mice after indicated treatments. e) Photos of tumors collected from mice after different treatments. f) H&E‐, Ki67‐, and TUNEL‐stained images of tumors after different treatments. Scale bars: 50 µM. Data are means ± SD, *n* = 5.

Encouraged by the in vitro antitumor efficacy and in vivo tumor accumulation of NTP@GOx, the efficacy of synergistic starvation/Type‐I PDT/chemotherapy was evaluated. As shown in **Figure** **5**a, the tumor growth of mice treated with NTP@GOx plus laser irradiation was significantly inhibited within 14 days compared with other treatments. Particularly, NTP@GOx+L group showed the highest tumor growth inhibition rate (98.0%), which was significantly higher than the control, NTP (4.2%), NTP+L (62.0%), and NTP@GOx (51.3%) groups (Figure 5b,c). Unexpectedly, the NTP+L group exhibited tumor inhibition effects, while its efficacy was weak at the cellular level. This is attributed to the significantly higher H_2_O_2_ levels in tumor tissues (10–100 µM) compared to in vitro cells.^[^ ^45^, ^46^ ^]^ The ex vivo tumor weight and tumor size indicated as photographs demonstrated that 60% of the tumors were completely eradicated and the rest were effectively suppressed in NTP@GOx+L group (Figure 5d,e), further confirming the robust tumor inhibition efficiency. In addition, the antitumor efficiency of NTP@GOx was assessed by hematoxylin and eosin (H&E), Ki67‐related antigen, and terminal transferase dUTP nick‐end labeling (TUNEL) staining of tumor sections after treatment at day 14. These results showed that significant growth inhibition (10% of Ki67 positive expression rate) and apoptosis (90% of TUNEL positive expression rate) occurred in tumor cells treated with NTP@GOx plus laser irradiation (Figure 5f; Figure S27, Supporting Information).

Importantly, there was no significant change in the body weight of mice during the treatment period (Figure S28, Supporting Information), indicating that NTP@GOx exhibited no acute toxicity. Besides, no significant abnormalities were observed in the H&E‐stained images of major organs (Figure S29, Supporting Information) and blood biochemical analysis (Figure S30, Supporting Information) of mice treated with NTP@GOx, further validating the good biocompatibility of NTP@GOx. NTP@GOx realizes carrier‐free delivery of NTP and GOx, which reduces the complexity and side effects associated with introducing and optimizing additional carriers.^[^ ^47^, ^48^, ^49^, ^50^ ^]^ Moreover, NTP@GOx can increase the drug loading capacity, thus improving the bioavailability of the loaded drugs. Most importantly, NTP@GOx can achieve more precise and controllable drug release, thereby improving therapeutic efficacy. Although 660 nm laser‐activated PDT exhibits low phototoxicity and minimal harm to normal tissues,^[^ ^51^ ^]^ its limited depth of penetration renders NTP@GOx more suitable for treatment of epidermal lesions and superficial tumors.

## 3 Conclusion

An activatable heterodimeric NTP prodrug‐GOx assembly (NTP@GOx) was developed for self‐boosting programmable release of therapeutic agents against tumors. The prodrug NTP, composed of ROS‐responsive TK‐linked NBS and PTX, exhibited a strong binding affinity to GOx (−8.1 kcal mol^−1^) due to their strong hydrophobic interaction, thus obtaining NTP@GOx assembly. On the one hand, NTP@GOx maximized the tumor accumulation of NTP. On the other hand, NTP@GOx can be degraded in TME, and restore the catalytic activity of GOx, thus performing glucose starvation therapy. The dual‐self‐boosting programmable release was achieved by the GOx catalysis‐triggered pH decrease, accelerating the dissociation of NTP@GOx, as well as the GOx catalysis‐triggered H_2_O_2_ generation to cleave the TK linker between NBS and PTX, expediting the release of NBS and PTX for Type‐I PDT and chemotherapy, respectively. In vitro cytotoxicity assays showed that NTP@GOx achieved high antitumor efficiency in the co‐presence of laser irradiation and glucose. Importantly, the 4T1 tumor‐bearing mice treated with NTP@GOx showed complete tumor eradication without side effects. Our findings indicate the as‐prepared NTP@GOx shows great potential for molecular imaging‐guided multimodal synergistic cancer therapy.

## 4 Experimental Section

### Materials

The chemicals used for the synthesis of NBS‐TK‐PTX (NTP) were supplied by Energy‐Chemical (Shanghai, China). Dihydrorhodamine 123 (DHR123), dihydroethidium (DHE), penicillin‐streptomycin, and fetal bovine serum (FBS) were purchased from ThermoFisher Scientific (Shanghai, China). Glucose oxidase (GOx) was supplied by Sigma Aldrich (Shanghai, China). Ethanol and dimethyl sulfoxide (DMSO) were supplied by J&K Scientific (Shanghai, China). The hydrogen peroxide (H_2_O_2_) assay kit was supplied by Beyotime (Shanghai, China).

### Synthesis of NBS‐TK

The ROS‐cleavable thioketal (TK) linker (300.00 mg, 1.19 mmol) was dissolved in anhydrous dimethylformamide (DMF, 10 mL), and then N‐Hydroxysuccinimide (NHS, 47.00 mg, 408.38 µmol) and 1‐(3‐Dimethylaminopropyl)‐3‐ethyl carbodiimide hydrochloride (EDCl, 100.00 mg, 639.99 µmol) were added to the above mixture under a nitrogen atmosphere. After stirring for 20 min, anhydrous DMF (2 mL) containing NBS^[^ ^52^ ^]^ (242.74 mg, 0.60 mmol) was added dropwise. The crude product was purified by silica gel column chromatography with CH_2_Cl_2_/CH_3_OH (10/2) to collect the desired NBS‐TK as a deep blue solid (150.00 mg, 39.10% yield). ^1^H NMR (600 MHz, DMSO‐d6) δ 8.94 (d, J = 8.1 Hz, 1H), 8.62 (d, J = 8.2 Hz, 1H), 8.01 (t, J = 5.6 Hz, 1H), 7.96–7.89 (m, 2H), 7.85–7.80 (m, 1H), 7.54 (s, 1H), 7.33 (s, 2H), 3.71 (t, J = 7.1 Hz, 2H), 3.64 (q, J = 13.8, 6.8 Hz, 4H), 3.14 (q, J = 12.6, 6.6 Hz, 2H), 2.74 (t, J = 7.4 Hz, 2H), 2.68 (t, J = 7.2 Hz, 2H), 2.35 (t, J = 7.4 Hz, 2H), 1.80–1.72 (m, 2H), 1.61–1.53 (m, 2H), 1.46 (s, 6H), 1.27 (t, J = 7.2 Hz, 2H), 1.22 (t, J = 7.1 Hz, 6H). HR‐MS (ESI): m/z calculated for C_33_H_43_N_4_O_3_S_3_^+^ = 639.24918, found m/z = 639.25110.

### Synthesis of NTP

Paclitaxel (PTX, 70.00 mg, 81.98 µmol) and NBS‐TK (150.00 mg, 234.41 µmol) were added into DMF (10 mL) solution, and then 4‐dimethylaminopyridine (DMAP, 54.00 mg, 442.00 µmol) and EDCl (90.00 mg, 575.99 µmol) were added and stirred for 48 h. The desired NTP was a deep blue solid (120.00 mg, 18.00% yield). ^1^H NMR (500 MHz, DMSO‐d6) δ 9.86 (s, 1H), 9.22 (d, J = 8.5 Hz, 1H), 9.00 (d, J = 8.2 Hz, 1H), 8.53 (d, J = 8.3 Hz, 1H), 8.05–7.90 (m, 5H), 7.88–7.84 (m, 3H), 7.75–7.72 (m, 1H), 7.68–7.65 (m, 2H), 7.59–7.52 (m, 2H), 7.48–7.40 (m, 8H), 7.19–7.16 (m, 1H), 6.28 (s, 1H), 5.81 (t, J = 8.8 Hz, 1H), 5.54 (t, J = 8.8 Hz, 1H), 5.41 (d, J = 7.2 Hz, 1H), 5.36 (d, J = 9.1 Hz, 1H), 4.91–4.89 (m, 2H), 4.62 (s, 1H), 4.12–4.07 (m, 1H), 4.03–3.98 (m, 2H), 3.71–3.64 (m, 6H), 3.57–3.56 (m, 1H), 3.37 (s, 2H), 3.17–3.13 (m, 2H), 2.72–2.67 (m, 6H), 2.33–2.30 (m, 3H), 2.22 (s, 3H), 2.09 (s, 3H), 1.78 (s, 6H), 1.59–1.53 (m, 2H), 1.49 (s, 3H), 1.39 (s, 6H), 1.23 (t, J = 7.1 Hz, 6H), 1.00 (d, J = 17.3 Hz, 6H). ^13^C NMR (126 MHz) δ 202.80, 171.34, 170.80, 170.11, 169.57, 169.24, 166.84, 165.68, 153.53, 151.34, 139.80, 137.70, 134.67, 133.87, 133.75, 130.40, 130.04, 129.18, 128.77, 128.12, 127.92, 105.87, 80.72, 77.18, 60.23, 57.88, 56.21, 53.26, 45.60, 43.41, 38.41, 35.72, 30.91, 30.88, 27.15, 26.80, 26.19, 26.14, 25.07, 23.05, 21.83, 21.24, 21.15, 14.56, 14.38, 13.12, 10.24. HR‐MS (ESI): m/z calculated for C_80_H_92_N_5_O_16_S_3_^+^ = 1474.56957, found m/z = 1474.57100.

### NTP Loading and Releasing Assays

5.00 mM of *β*‐Mercaptoethanol (*β*‐Me) and tris(hydroxymethyl)aminomethane (Tris) buffer solution (10 mL) were added in a 20 mL glass vial. After stirring for 5 min, 20.00 mg of GOx (2.00 mg mL^−1^) was added and stirred for another 10 min. Then, 0.05 mg mL^−1^ of NTP was added into the above mixture, which continued to stir at 37 °C for 10 min. The excess NTP was removed by centrifugation at 7500 rpm, and the desired product NTP@GOx was collected. For NTP releasing confirmation, the NTP@GOx (1 mL, concentration of NTP@GOx / NTP: 5.73 / 0.25 mg mL^−1^) was added into a dialysis bag (MWCO = 14 kDa), and then put into the beaker containing PBS (5 mL) at pH 5.0, 5.5, 6.0, 6.5, 7.0, and 7.4, respectively. After 1 h, 5 mL of the sample was drawn, and the absorption intensity was measured to detect the concentration of released NTP.

### PTX and NBS Releasing Assays

For PTX and NBS releasing confirmation, the NTP@GOx was loaded in a dialysis bag and then the bag was immersed in PBS (pH 7.4 or 5.0, with 0.1% v/v Tween‐80) with or without 10 mM glucose. 1 mL of PBS was collected and supplemented at different time points. The concentrations of PTX and NBS were measured using HPLC. Flow phase: 70% acetonitrile (containing 1% formic acid) and 30% ultrapure water (containing 5% acetonitrile and 1% formic acid); flow rate: 1.0 mL min^−1^; loading volume: 10 µL; elution time: 6.5 min.

### Molecular Docking Calculation

AutoDocking Vina Tool‐1.5.6 was used to simulate the docked confirmation of GOx with NTP and NBS, respectively. In docking simulations, the binding free energies and binding sites of NTP with active moiety of GOx were analyzed. Then, the crystal structure of GOx was obtained from RCSB PDB (1CF3) and set the parameters as solvent removal, energy refinement, and hydrogen addition for stable configuration during AutoDocking. The grid box for envelopment active moiety was defined with a spacing of 10 Å and the dimensions were at 72 × 66 × 54 Å points. The theoretical calculations were repeated several times to confirm the reproducibility of docking simulation, and the highest binding affinity of docked models was represented in this work.^[^ ^42^ ^]^

### Catalytic Activity Detection

The time‐dependent (0, 1, 2, 5, 10, 20, 30, 40, 50, and 60 min) catalytic activity of NTP@GOx/dissociated‐NTP@GOx with glucose solution (5 mM) was analyzed. Then, the catalytic activity of dissociated‐NTP@GOx and GOx (concentration of GOx: 10 µg mL^−1^, concentration of NTP@GOx: 10.45 µg mL^−1^) was measured by incubating with various concentrations of glucose (0, 0.01, 0.05, 0.1, 0.5, 1, 2, 5, and 10 mM) for 1 h.

### O_2_^−•^ Detection in Solution

Dihydrorhodamine 123 (DHR123) was used for O_2_^−•^ detection in NTP@GOx and NTP. In detail, NTP@GOx (concentration of NTP@GOx: 1.15 µg mL^−1^, concentration of NTP: 50 ng mL^−1^) or NTP (50 ng mL^−1^) were added into PBS (pH 5.0), and then glucose (10 mM) and DHR123 (5 µM) were added into the above solution. Vitamin C was used for O_2_^−•^ scavenging. Similarly, this experiment was also conducted for control groups under different conditions. Finally, the emission spectra of the different groups were observed.

### In Vitro ROS Detection

4T1 cells were incubated with NTP@GOx (0, 0.125, 0.25, 0.5, and 1 µg mL^−1^) with or without 10 mM of glucose, and then incubated with serum‐free dulbecco's modified eagle medium (DMEM) containing 10.00 µM of DCFH‐DA for another 30 min. The green DCFH‐DA emission was observed immediately by using high‐content screening (PerkinElmer, America).

### Intracellular O_2_^−•^ Detection

Hypoxic or normoxic 4T1 cells were incubated with NTP@GOx (concentration of NTP@GOx: 1.15 µg mL^−1^, concentration of NTP: 50.00 ng mL^−1^) or NTP (50.00 ng mL^−1^) for 1 h, and then incubated with 5.00 µM of DHE for another 30 min. Next, the cells were irradiated by a 660 nm laser (0.2 W cm^−2^) for 5 min. The red FL signal of DHE was observed immediately by using high‐content screening (PerkinElmer, America).

### In Vitro Cellular Uptake

4T1 cells were incubated with NTP@GOx (concentration of NTP@GOx: 1.15 µg mL^−1^) or NTP (50 ng mL^−1^). Next, fluorescence images were recorded at different periods (2, 4, 6, 8, 10, 12, 14, 16, 18, 20, 22, and 24 h) with high‐content screening (PerkinElmer, America) under 660 nm excitation wavelength and 700 nm emission wavelength, respectively.

### Lysosomal/Mitochondrial Co‐localization Aassays

4T1 cells were incubated with NTP@GOx (concentration of NTP@GOx: 1.15 µg mL^−1^, concentration of NTP: 50 ng mL^−1^) for 4 h, and then stained with Hoechst probe (10.00 µM) for 10 min. Next, the cells were incubated with a lysosomal probe (Lyso Tracker, 100.00 nM) and mitochondrial probe (Mito Tracker, 100.00 nM) for another 30 min, respectively. Finally, the cells were observed immediately by using confocal laser scanning microscope (LSM880, Carl Zeiss, Germany). The excitation/emission wavelengths of Lyso Tracker, Mito Tracker, and NTP were 570/590, 490/520, and 640/680 nm, respectively.

### In Vitro Cytotoxicity Assays

Hypoxic (2% O_2_) or normoxic 4T1 cells were pretreated with or without 2.00 mM of glucose, and NTP@GOx or NTP was added with different concentrations. Then, cells were conducted PDT (660 nm laser; 0.2 W cm^−2^, 5 min) and incubated for another 16 h. Next, removed the original medium and added 100 uL of MTT (0.50 mg mL^−1^) solution containing DMEM, after 4 h incubation, the media was replaced with DMSO to complete the staining of blue formazan. Eventually, cell viability was measured by using a Synergy H1 microplate reader (BioTek, America).

### Live/Dead Cell Staining

4T1 cells were treated with 7 groups as follows: group 1, control; group 2, incubated with 50.00 ng mL^−1^ of NTP (NTP) for 24 h; group 3, incubated with 50.00 ng mL^−1^ of NTP for 8 h and irradiated with 660 nm laser (NTP+L); group 4, incubated with 1.15 µg mL^−1^ of NTP@GOx for 24 h (NTP@GOx); group 5, incubated with 1.15 µg mL^−1^ of NTP@GOx and 2 mM of glucose for 24 h (NTP@GOx+G); group 6, incubated with 1.15 µg mL^−1^ of NTP@GOx for 8 h and irradiated with 660 nm laser (NTP@GOx+L); group 7, incubated with 1.15 µg mL^−1^ of NTP@GOx and 2 mM of glucose for 8 h, and irradiated with 660 nm laser (NTP@GOx+G+L). The light dose was 0.6 W cm^−^^2^, with an irradiation duration of 5 min. All laser‐irradiated cells were incubated for another 16 h. After the above treatments, all groups were stained with CA/PI to examine FL signals of cells.

### Tumor Model Preparation and In Vivo Tumor Imaging

All procedures were carried out according to the regulations of the Animal Ethical and Welfare Committee of Shenzhen University (AEWC‐SZU). To prepare the subcutaneous xenograft model, 1 × 10^6^ 4T1 cells were injected subcutaneously into the right flank of the mice (16–20 g). NTP@GOx (dose of NTP: 0.50 mg kg^−1^) or NTP (dose: 0.50 mg kg^−1^) were injected intravenously into each tumor‐bearing BALB/c nude mice. Then, the in vivo/ex vivo FL images were acquired by the IVIS Spectrum system (PerkinElmer, America).

### In Vivo Tumor Hypoxia Detection

The percentage of tumor O_2_ level was analyzed by PA imaging. O_2_ percentage in 4T1 tumor‐bearing nude mice either treated with NTP@GOx or NTP was measured at different time points (0, 1, 2, 4, 6, and 12 h). Different colors in PA images represent B‐mode (grey color), OxyHb (red color), and DeoxyHb (blue color), respectively. The degree of tumor blood O_2_ saturation in each mouse was measured on the Vevo LAZR‐X system (VisualSonics, America).

### In Vivo PDT Treatment

To evaluate the multimodal treatment efficacy of NTP or NTP@GOx, tumor‐bearing mice were divided into 6 groups: 1) Saline, 2) Saline + 660 nm laser irradiation, 3) NTP (dose: 0.5 mg kg^−1^), 4) NTP (dose: 0.5 mg kg^−1^) + 660 nm laser irradiation, 5) only NTP@GOx (dose of NTP: 0.5 mg kg^−1^), and 6) NTP@GOx (dose of NTP: 0.5 mg kg^−1^) + 660 nm laser irradiation. One dose of i.v. injection was applied for all groups of mice on day 1. For laser treatment, a 660 nm laser at 0.2 W cm^−2^ power density was applied for 10 min.

### Statistical Analysis

Data are expressed as means ± standard deviation (SD). Differences between the two groups were evaluated via an unpaired two‐tailed Student's *t*‐test, whereas comparisons across multiple groups were performed using ANOVA. A *p*‐value of less than 0.05 was deemed statistically significant, denoted as follows: ^*^ *p* < 0.05, ^**^ *p* < 0.01, ^***^ *p* < 0.001; n.s. indicates non‐significance.

## Conflict of Interest

The authors declare no conflict of interest.

## Author Contributions

S.J. and B.G. contributed equally to this work. J.L. conceived the concept and supervised the project. S.J. and B.G. performed the experiments and all measurements. J.Z., S.L., Y.Z., and T.H. verified the performed the calculations. S.J. and B.G. wrote the original draft of the manuscript and J.L., P.H., O.T., and F.H., revised the manuscript. All authors discussed the results and commented on the manuscript.

## Supporting information



Supporting Information

## Data Availability

Research data are not shared.
