# Supplementary material for: Self‐Boosting Programmable Release of Multiple Therapeutic Agents by Activatable Heterodimeric Prodrug‐Enzyme Assembly for Antitumor Therapy
Source: Adv Sci (Weinh). 2024 Nov 21;12(2):2409960. doi: 10.1002/advs.202409960 (PMC11727268; doi:10.1002/advs.202409960)

Supporting Information

Self-Boosting Programmable Release of Multiple Therapeutic Agents by Activatable Heterodimeric Prodrug-Enzyme Assembly for Antitumor Therapy

Shanshan Jiang,^†[a, b]^ Bhaskar Gurram,^†[a, c]^ Junfei Zhu,^[a]^ Shan Lei,^[a]^ Yifan Zhang,^[a]^ Ting He,^[a]^ Oya Tagit,^[c]^ Hui Fang,^[b]^ Peng Huang^[a]^ and Jing Lin*^[a]^

[a] Dr. S. Jiang, Dr. B. Gurram, Dr. S. Lei, Dr J. Zhu, Dr. Y. Zhang, Dr. H. Ting, Prof. P. Huang and Prof. J. Lin

Marshall Laboratory of Biomedical Engineering, International Cancer Center, Laboratory of Evolutionary Theranostics, School of Biomedical Engineering,

Shenzhen University Medical School, Shenzhen University, Shenzhen 518055, China

[b] Dr. S. Jiang, Prof. H. Fang

Nanophotonics Research Center, Shenzhen Key Laboratory of Micro-Scale Optical Information Technology, Institute of Microscale Optoelectronics, Shenzhen University, Shenzhen 518060, China

[c] Dr. B. Gurram, Prof. O. Tagit

Department of BioInterfaces, Institute for Chemistry and Bioanalytics, School of Life Sciences, FHNW University of Applied Sciences and Arts Northwestern Switzerland, 4132 Muttenz, Switzerland

[†] These authors contributed equally to this work.

E-mail: jingl@szu.edu.cn

Supporting Figures


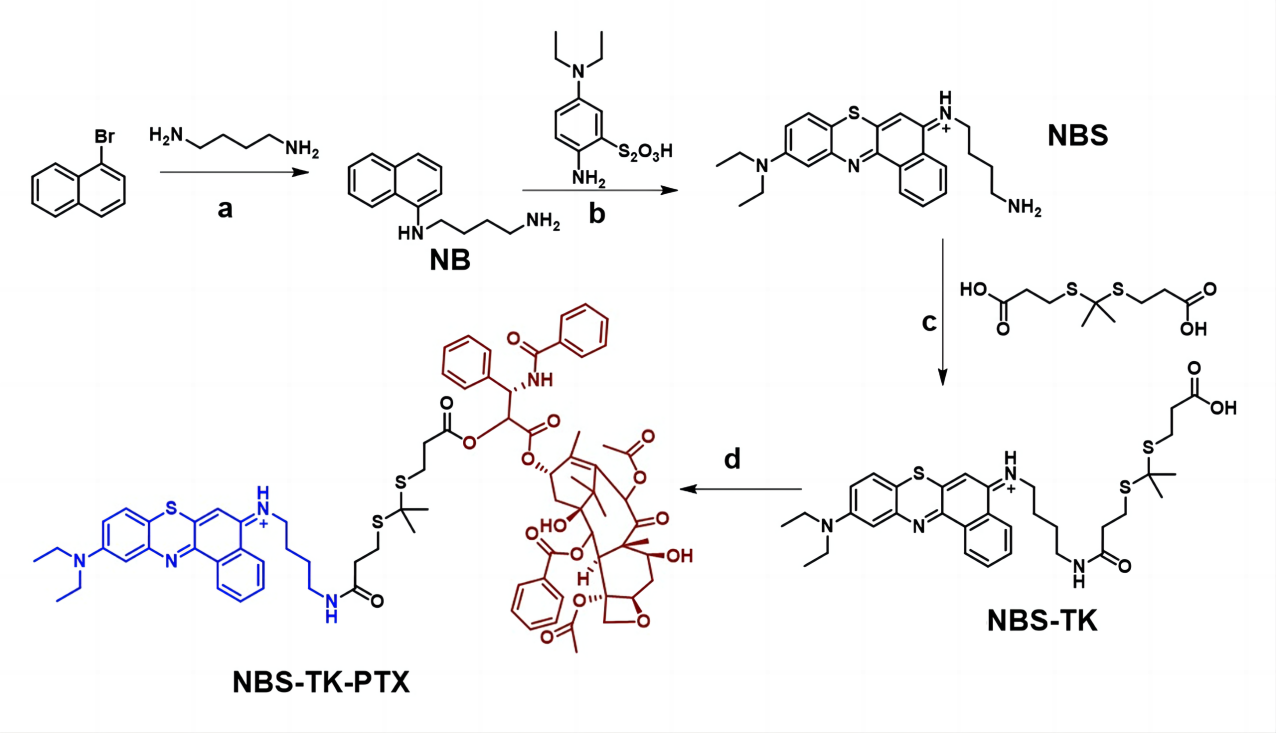


**Figure S1.** Synthesis route of NBS-TK-PTX (NTP).


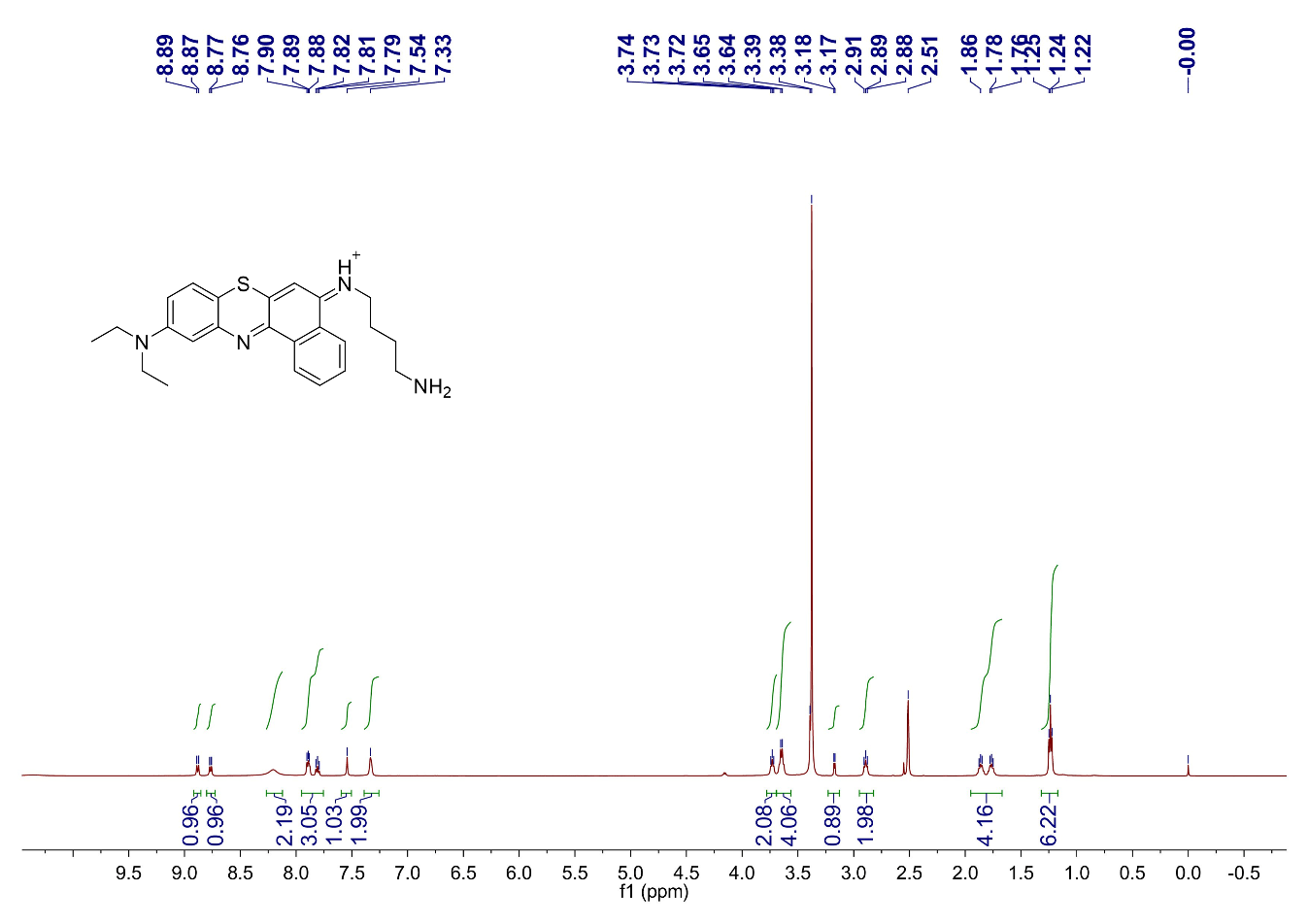


**Figure S2.** The ^1^H NMR spectrum of NBS (DMSO-d6).


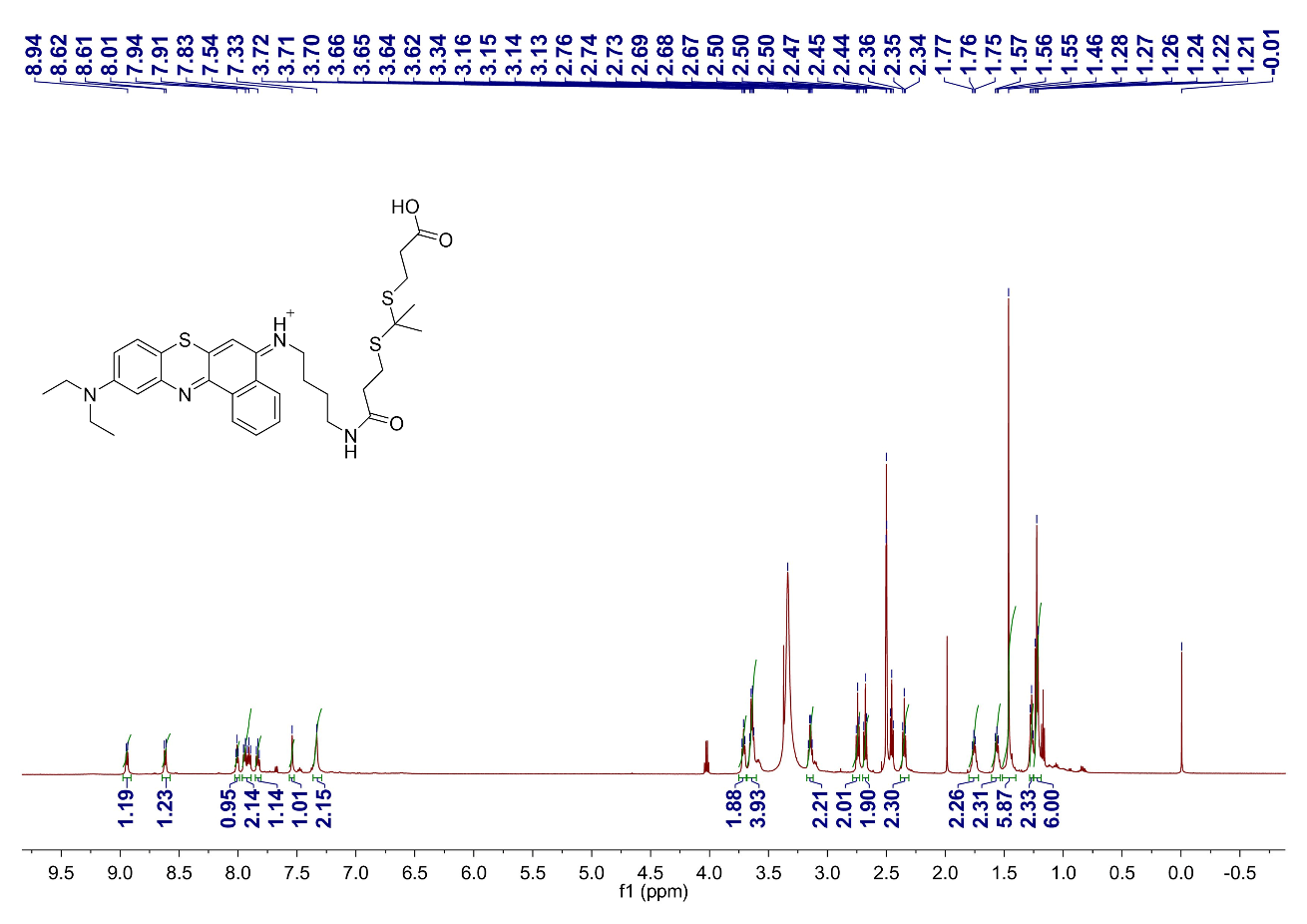


**Figure S3.** The ^1^H NMR spectrum of NBS-TK (DMSO-d6).


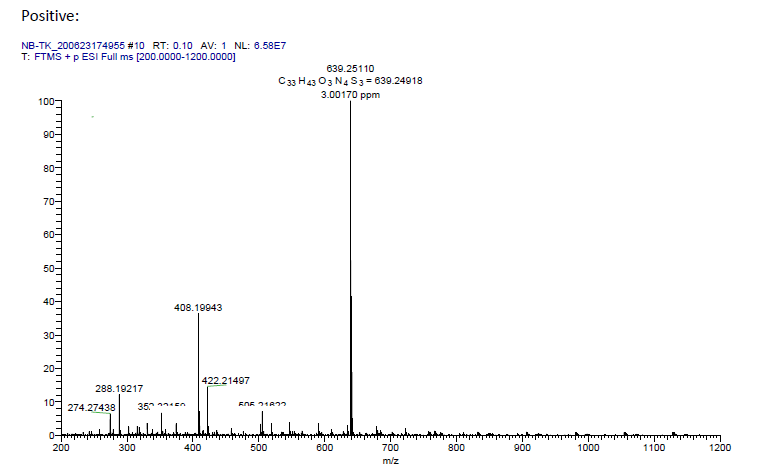


**Figure S4.** The HR-MS spectrum of NBS-TK.


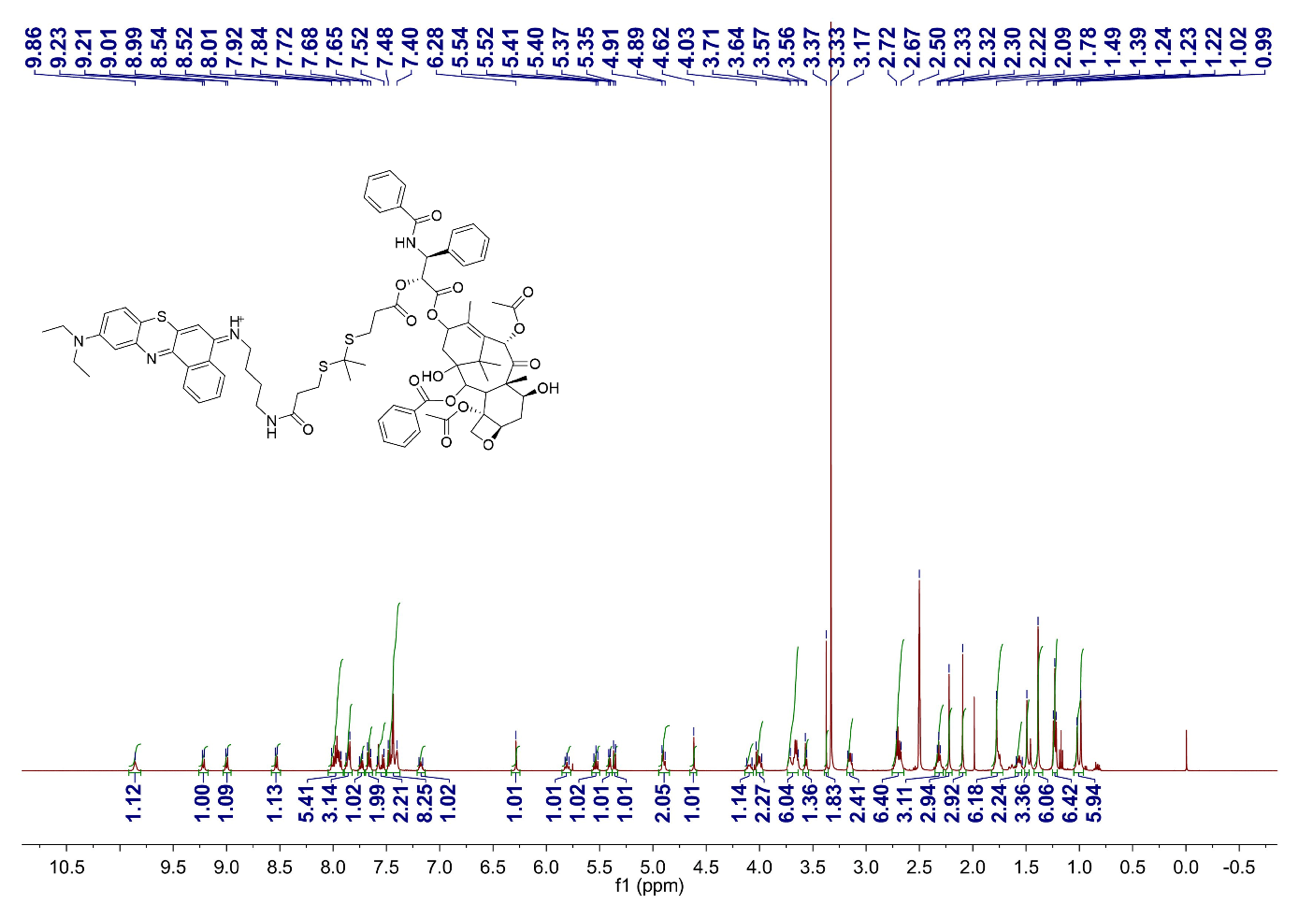


**Figure S5.** The ^1^H NMR spectrum of NTP (DMSO-d6).


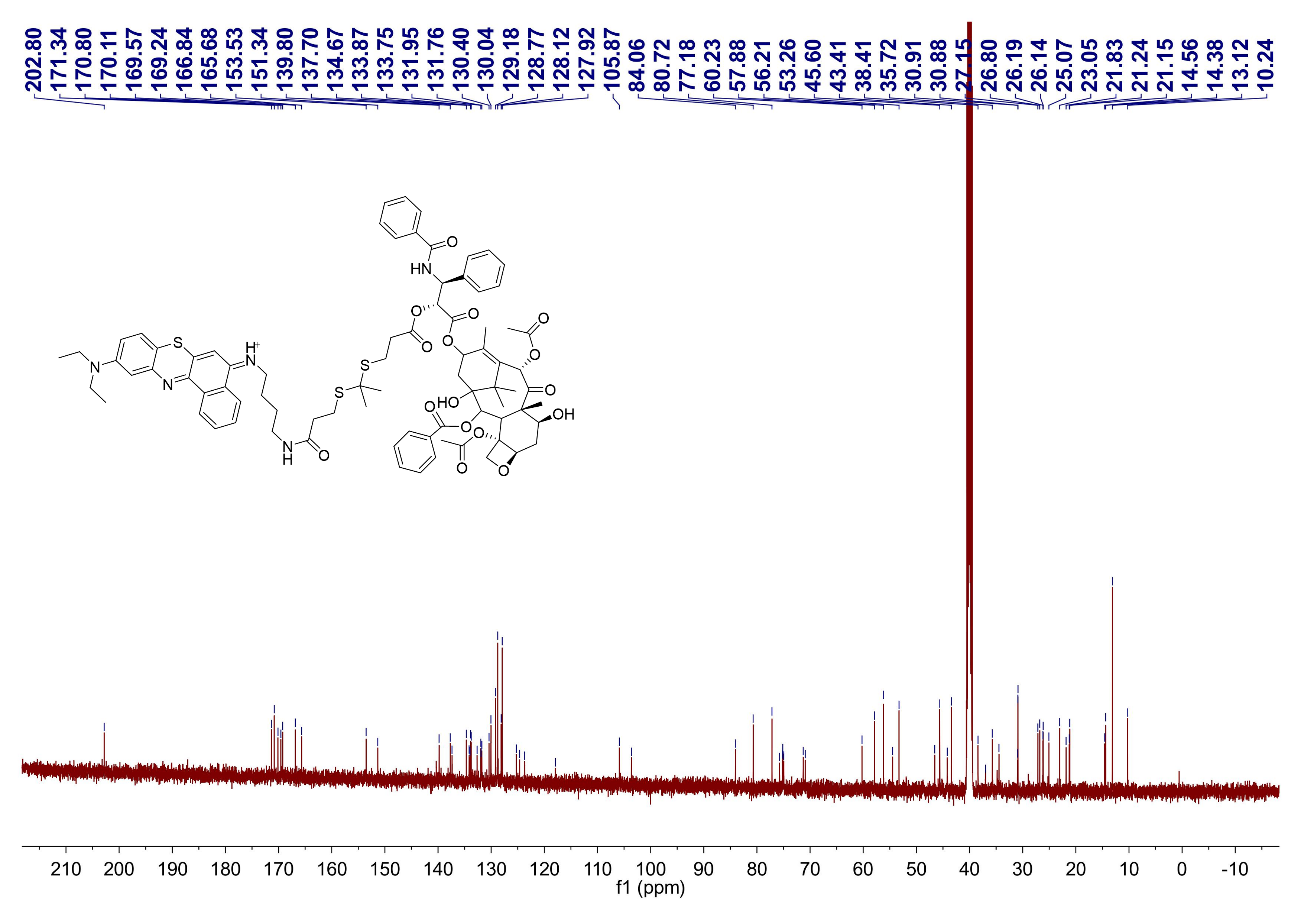


**Figure S6.** The ^13^C NMR spectrum of NTP (DMSO-d6).


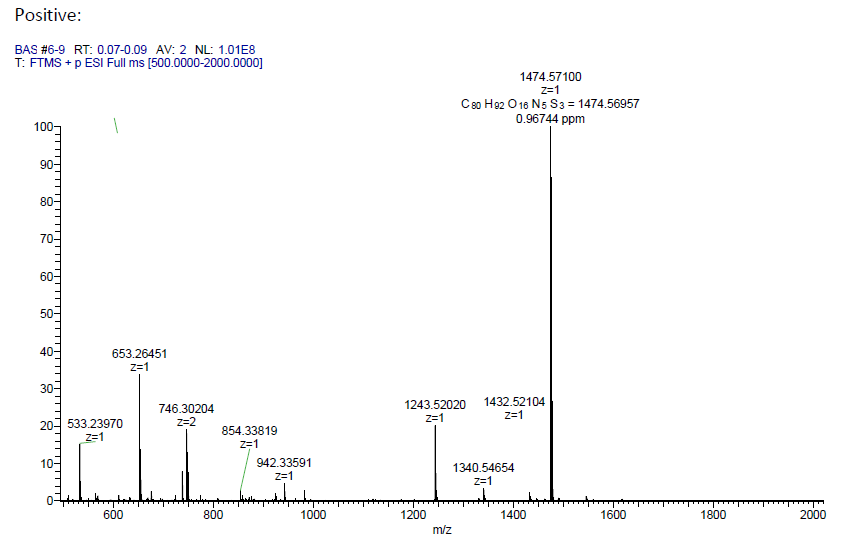


**Figure S7.** The HR-MS spectrum of NTP.


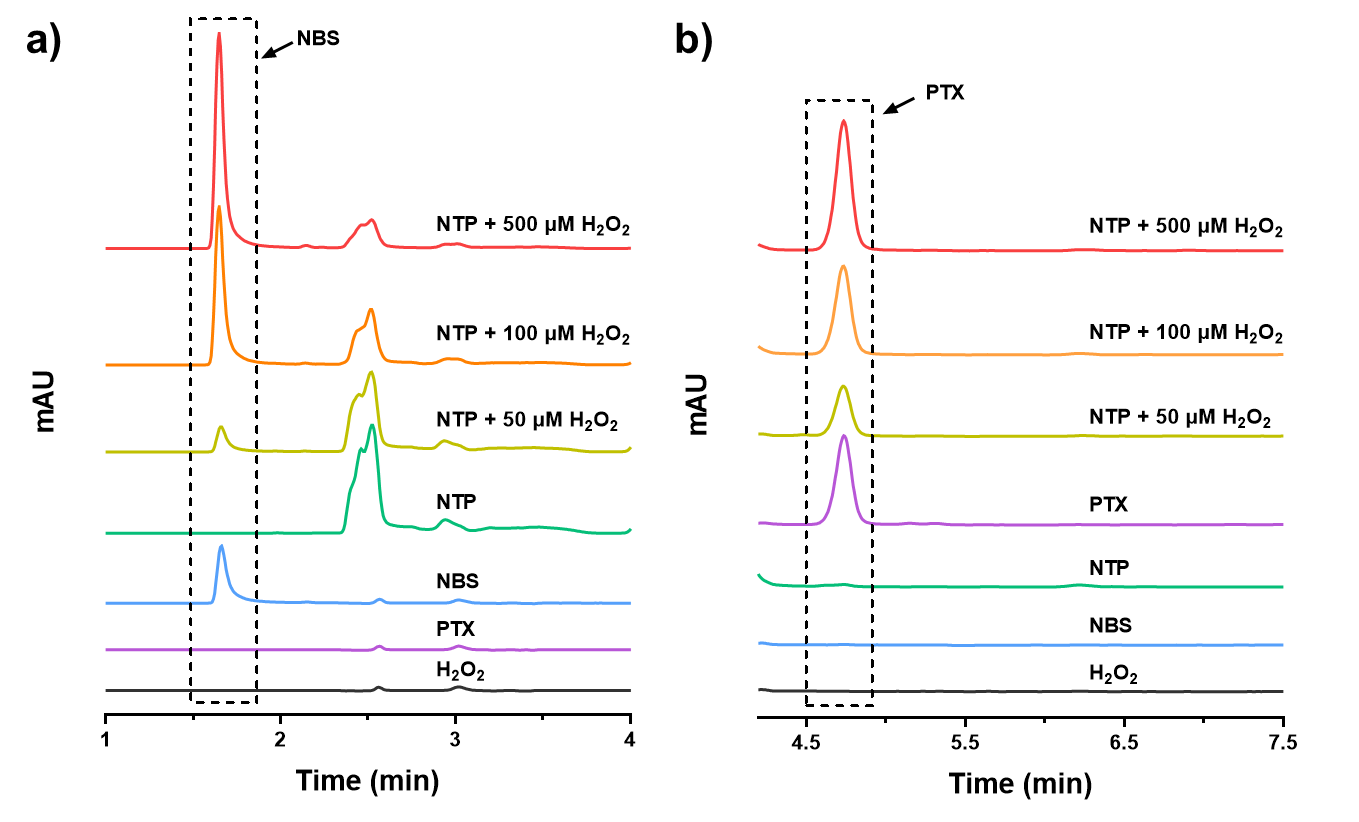


**Figure S8.** Comparative high-performance liquid chromatography (HPLC)-UV/DAD chromatographic profiles of a) NBS and b) PTX released from NTP in response to H_2_O_2_. Flow phase: 70% acetonitrile (containing 1% formic acid) and 30% ultrapure water (containing 5% acetonitrile and 1% formic acid).


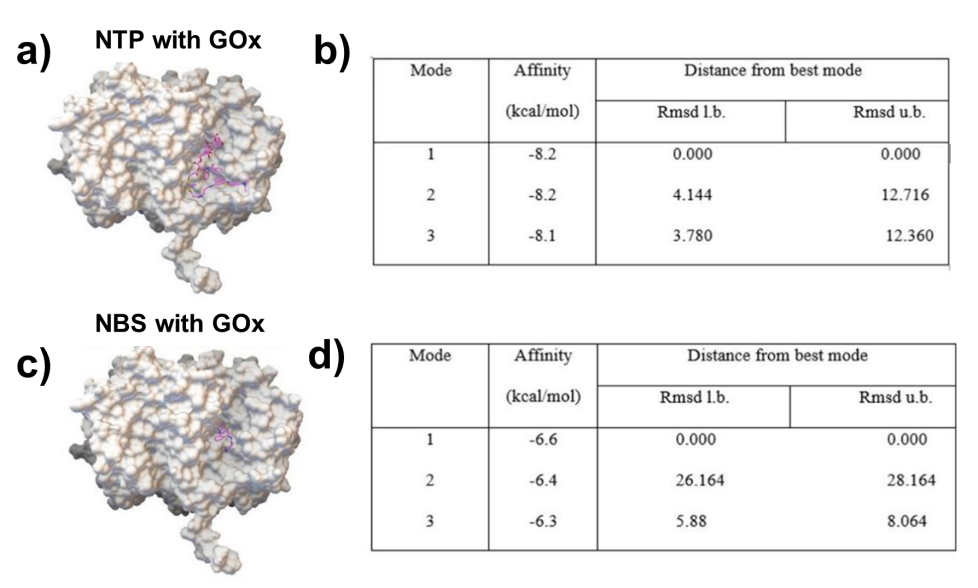


**Figure S9.** Binding affinity calculated by molecular docking vina. a, b) NTP with GOx (binding affinity: -8.2 to -8.1 kcal mol^-1^). c, d) NBS with GOx (binding affinity: -6.6 to -6.3 kcal mol^-1^).


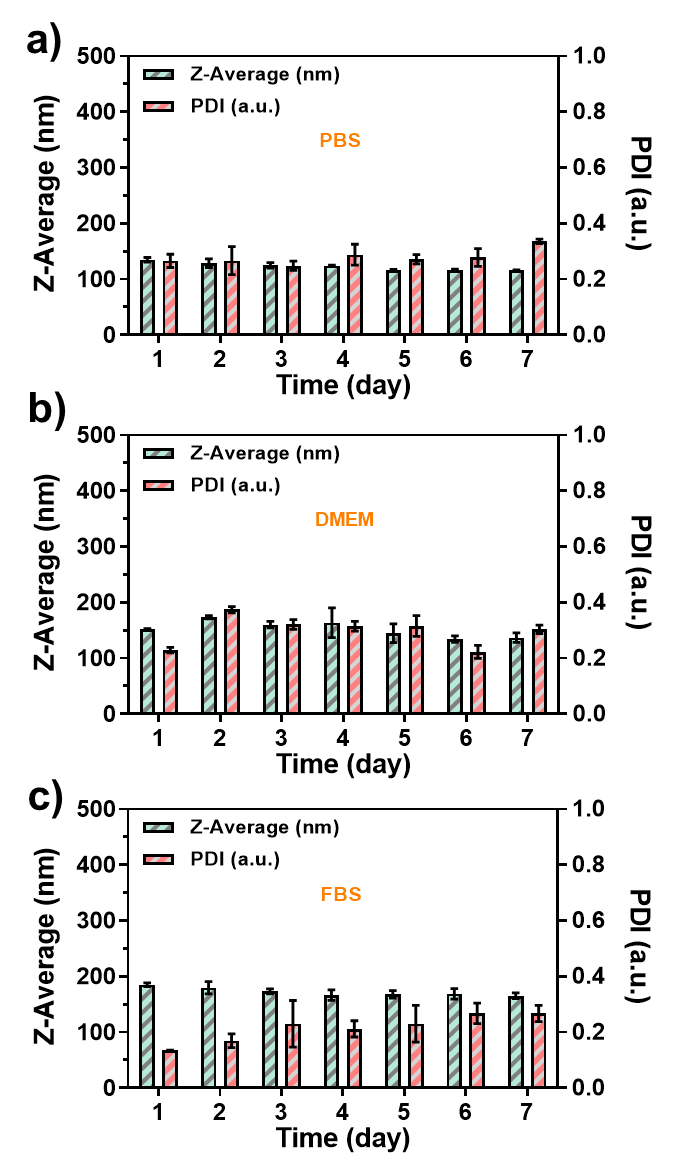


**Figure S10.** Hydrodynamic diameter and polydispersity index (PDI) of NTP@GOx in a) PBS, b) DMEM, and c) FBS during 7 days storage at 4 °C. Data are means ± SD, *n* = 3.


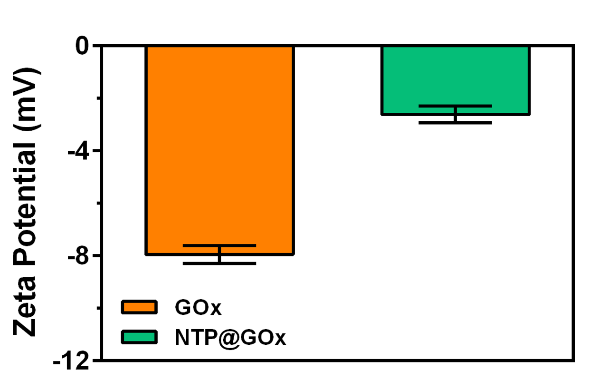


**Figure S11.** Zeta potentials of NTP@GOx and GOx in PBS. Data are means ± SD, *n* = 3.


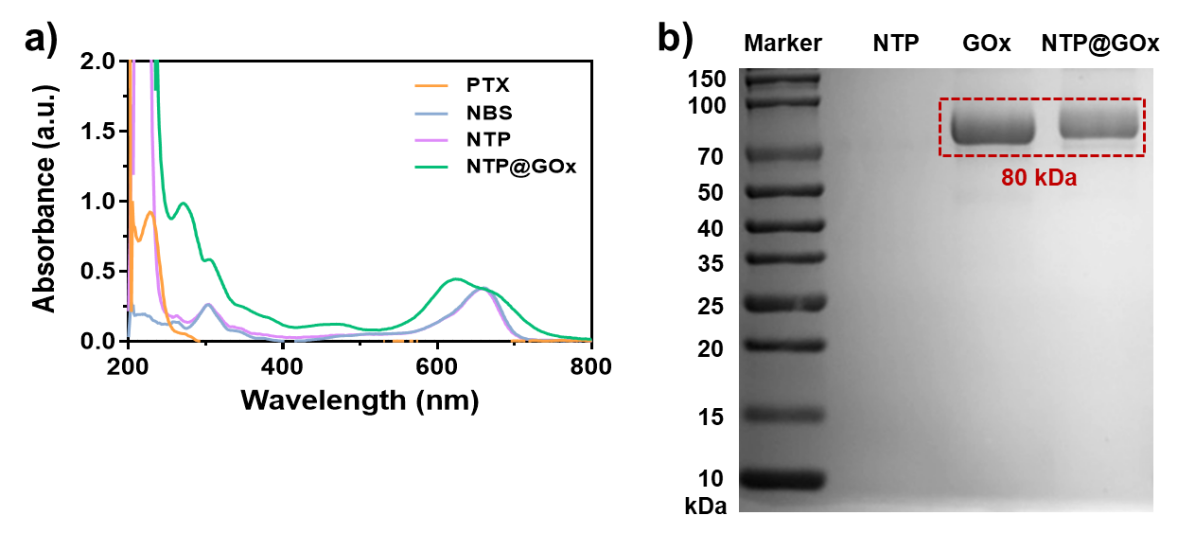


**Figure S12.** a) The absorption spectra of PTX, NBS, NTP, and NTP@GOx, respectively. b) Sodium dodecyl sulfate-polyacrylamide gel electrophoresis (SDS-PAGE) diagram of NTP, GOx, and NTP@GOx.


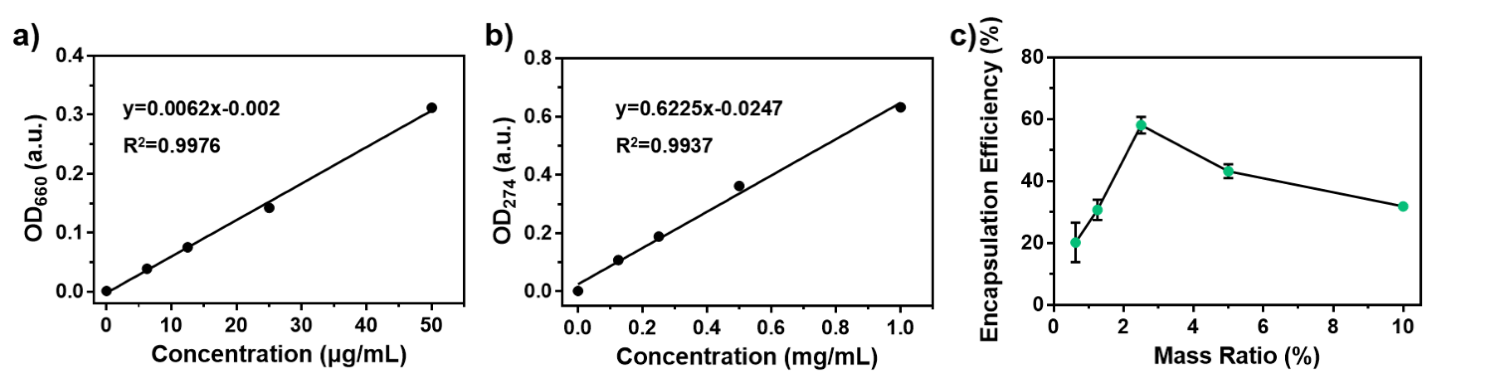


**Figure S13.** NTP and GOx encapsulation. a) Linear fit of the standard curve of optical density (OD) at 660 nm (OD_660_) to different concentrations of NTP. b) Linear fit of the standard curve of OD at 274 nm (OD_274_) to different concentrations of GOx. c) Encapsulation efficiency of NTP@GOx for different mass ratios of NTP to GOx. Data are means ± SD, *n* = 3.


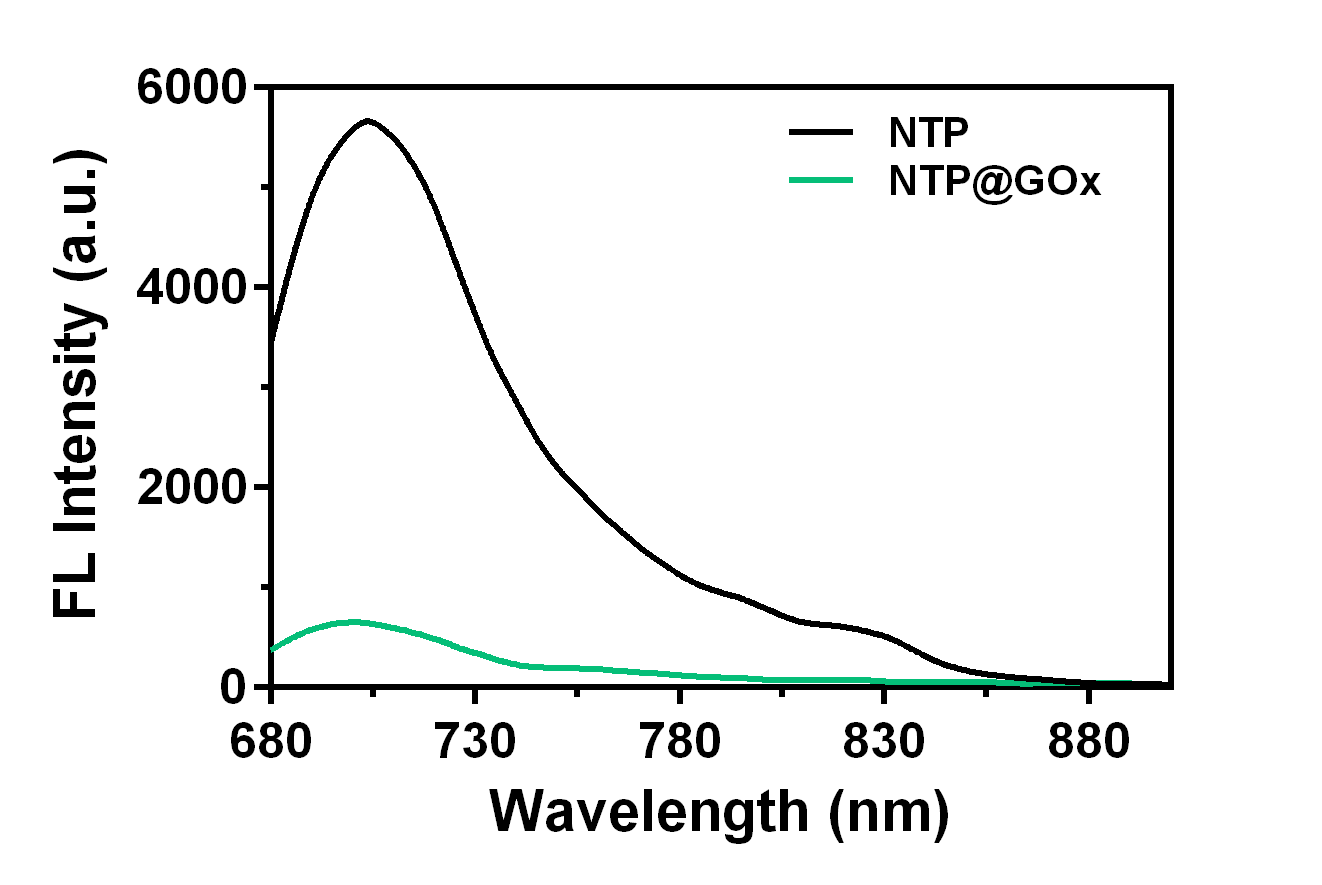


**Figure S14.** The FL spectra of NTP and NTP@GOx at same NTP concentration.


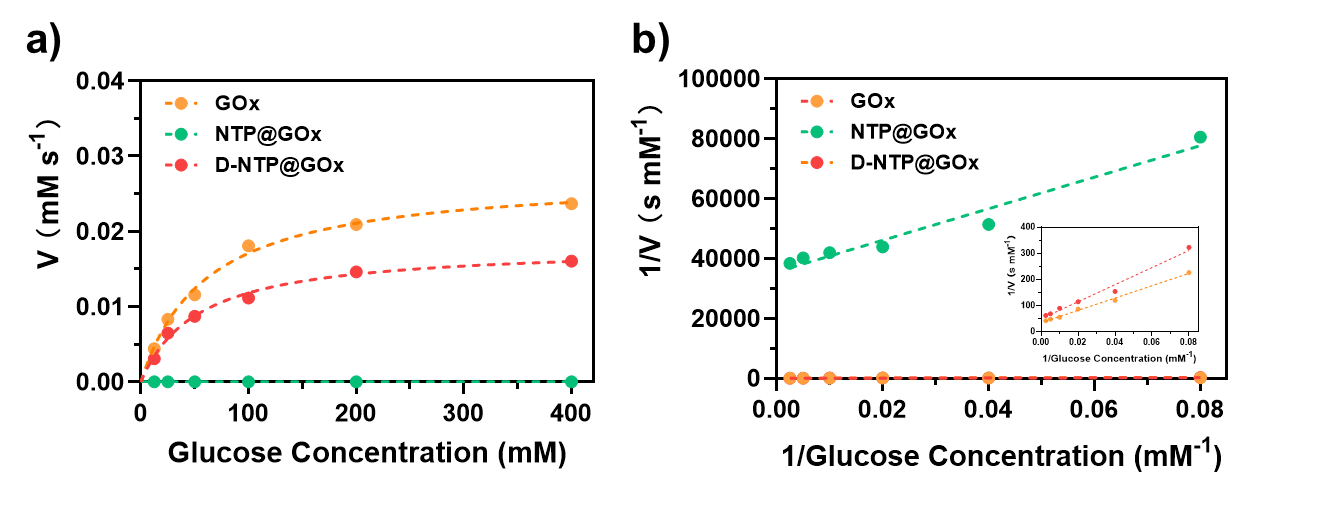


**Figure S15.** Kinetic analysis of GOx, NTP@GOx and D-NTP@GOx. a) Michaelis-Menten kinetics plot. V represented the initial reaction rate. b) Double reciprocal (Lineweaver-Burk) plot of the initial reaction rate versus glucose concentration.


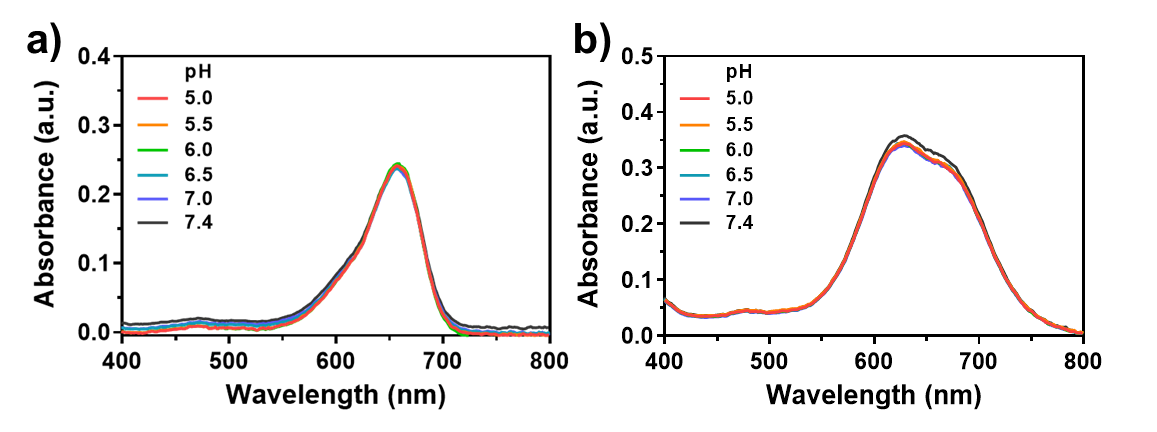


**Figure S16.** The absorption spectra of NTP in a) ethanol and b) PBS at different pH.

**Figure S17.** NBS release behaviors of NTP@GOx in different solutions. G: glucose. Data are means ± SD, *n* = 3.


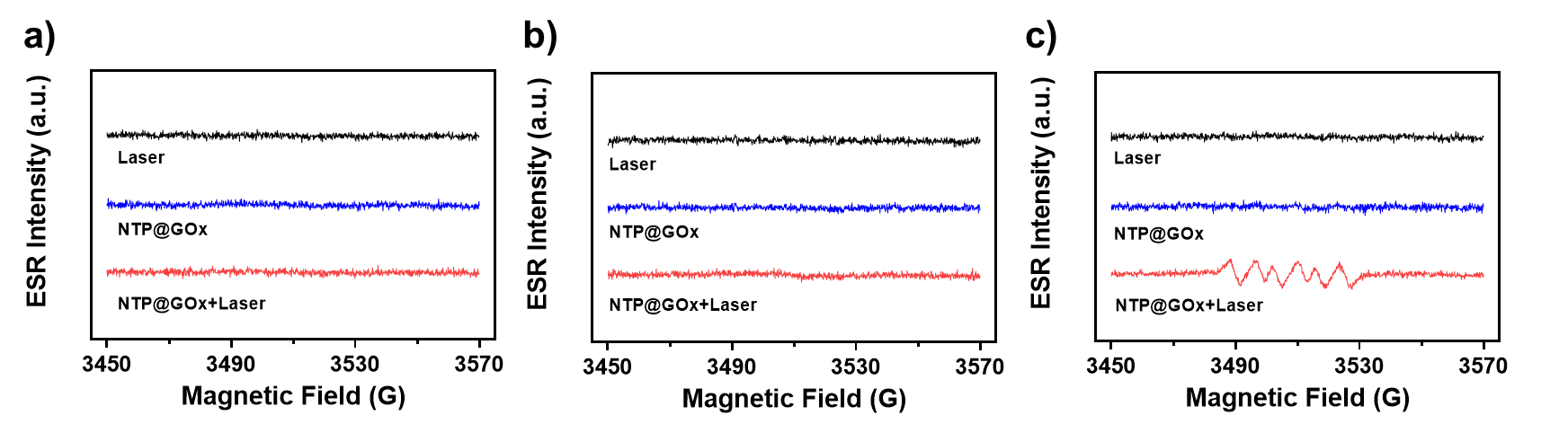


**Figure S18.** The electron spin resonance (ESR) spectra of different groups (Laser, NTP@GOx, or NTP@GOx+Laser) incubated with a) 5,5-Dimethyl-1-pyrroline N-oxide (DMPO) in H_2_O solution for •OH characterization, b) 2,2,6,6-Tetramethylpiperidine (TEMP) in H_2_O solution for ^1^O_2_ characterization and c) DMPO in MeOH solution for O_2_^-•^ characterization. NTP@GOx: pretreated with acidic PBS buffer (pH 5.0) and glucose (10 mM).


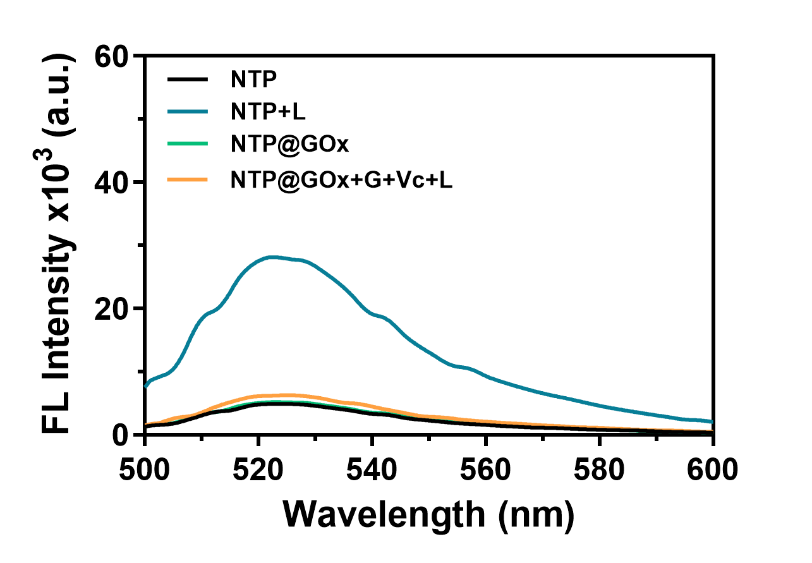


**Figure S19.** The FL spectra of DHR123 with different treatments at pH 5.0. G: 10 mM of glucose, L: 660 nm laser irradiation.


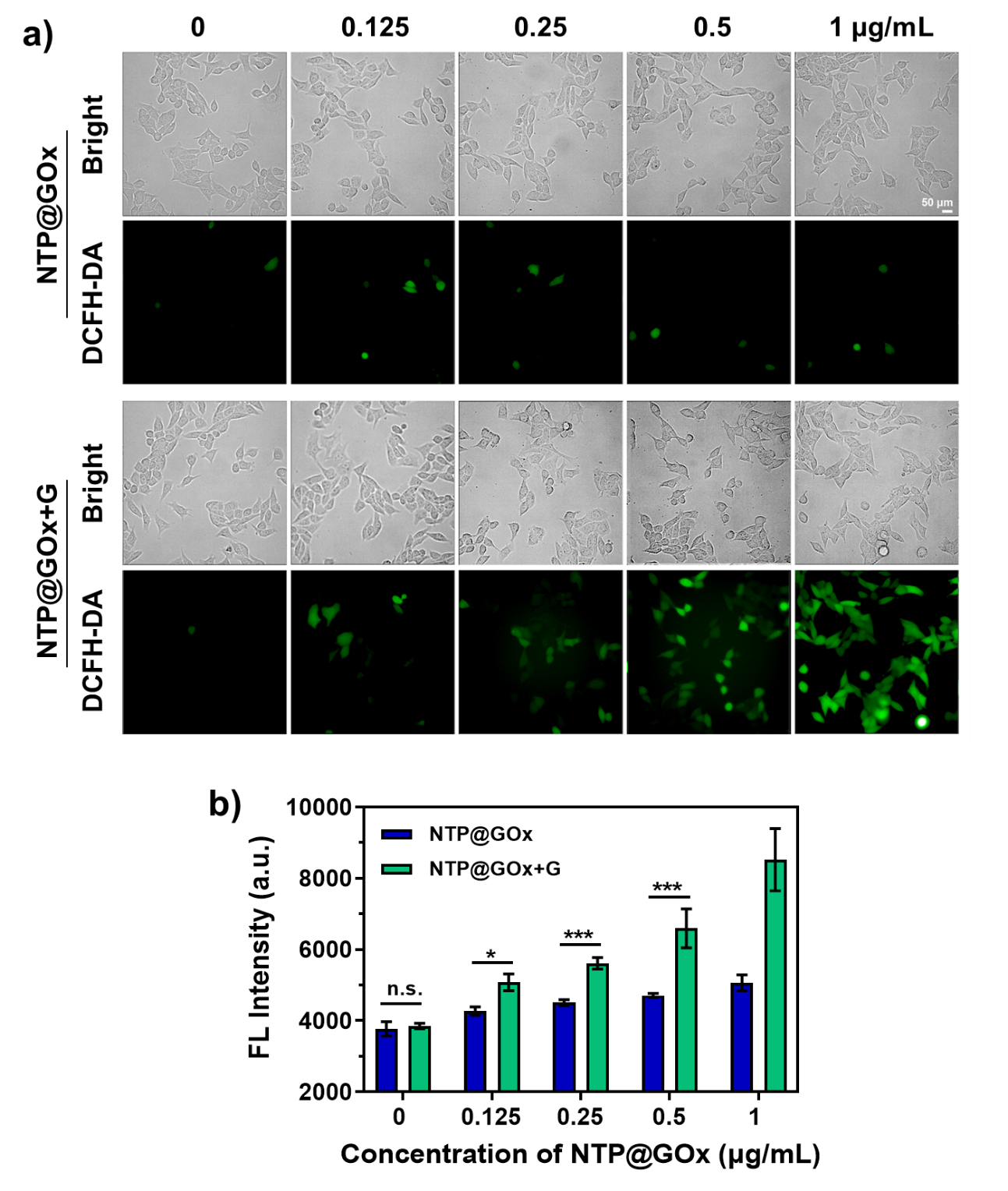


**Figure S20.** a) 2',7'-dichlorodihydrofluorescein diacetate (DCFH-DA)-stained images of 4T1 cells in glucose-free and glucose-containing conditions with different treatments. Scale bar: 50 μm. b) FL intensity of DCFH-DA in 4T1 cells after different treatments. Data are means ± SD, *n* = 3.


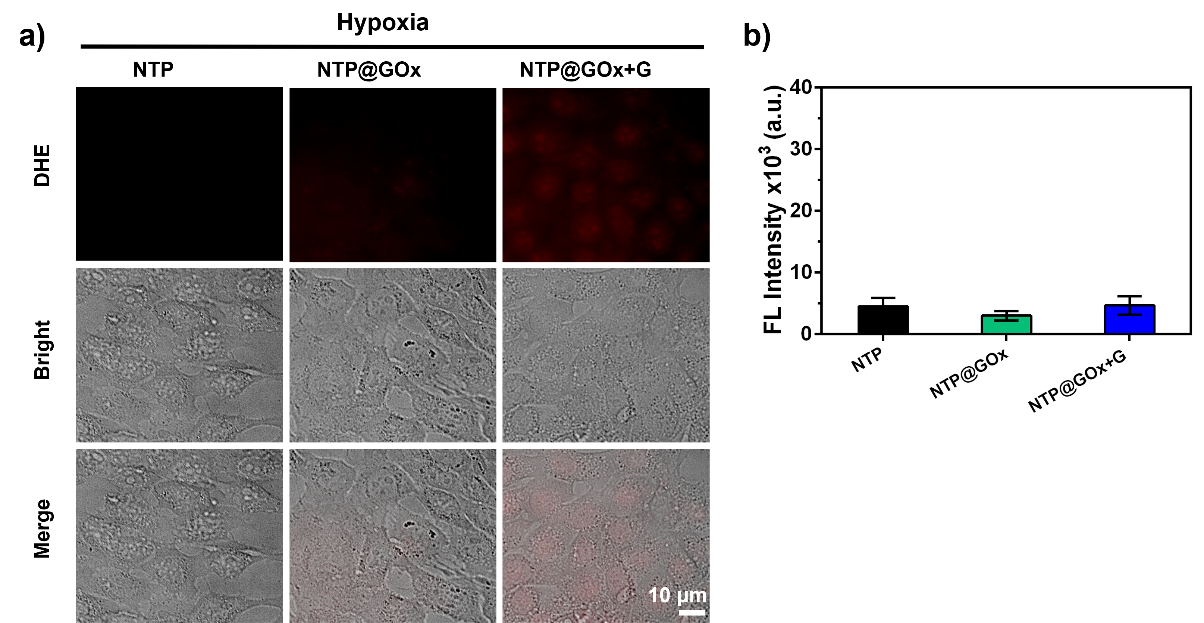


**Figure S21.** a) DHE stained images of 4T1 cells with different treatments under hypoxic conditions. Scale bar: 10 μm. b) FL intensity of DHE in 4T1 cells after different treatments under hypoxic conditions. Data are means ± SD, *n* = 4-6.


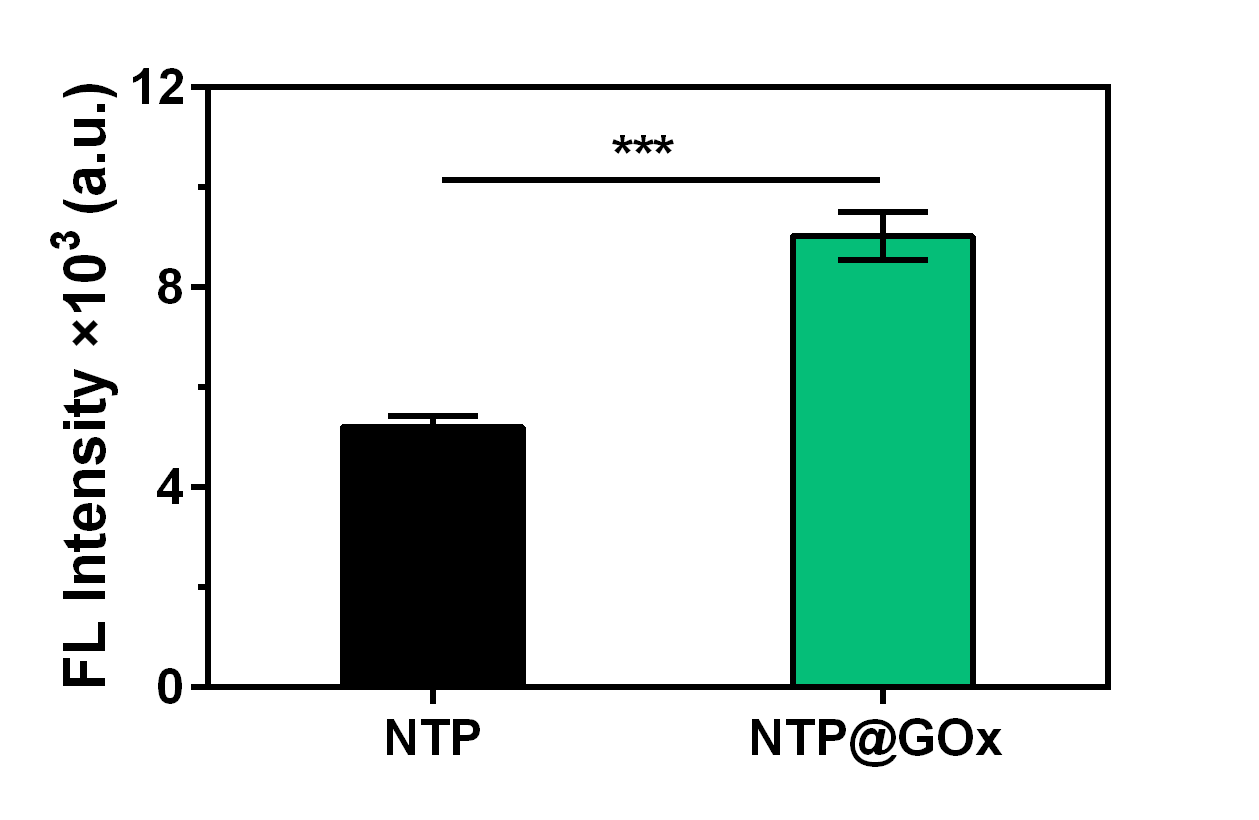


**Figure S22.** FL intensity of NTP and NTP@GOx in 4T1 cells, respectively. Data are means ± SD, *n* = 3.


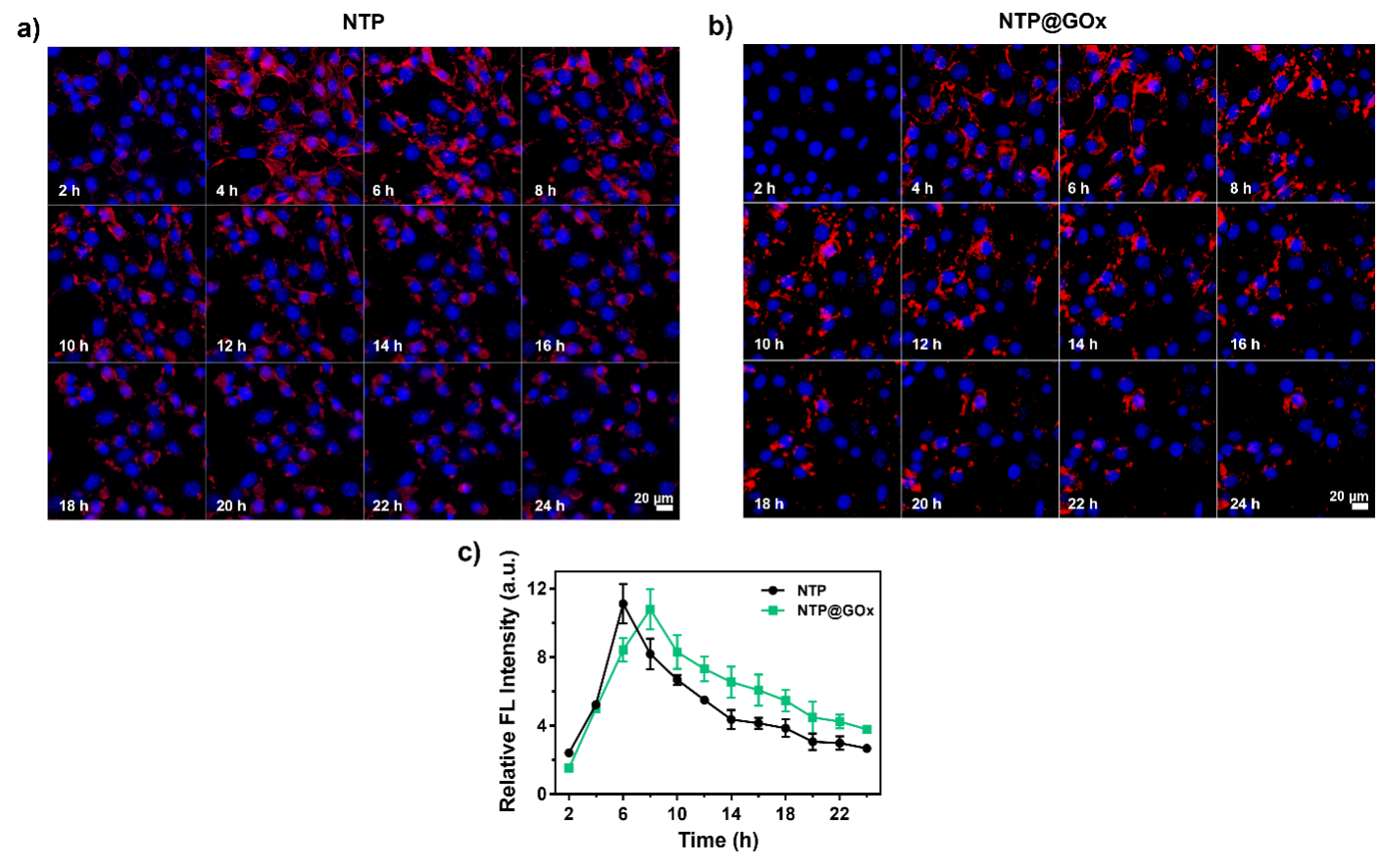


**Figure S23.** Assessment of cellular uptake efficiency. a) Hoechst and NTP co-stained images of 4T1 cells collected from 2 to 24 h. Scale bar: 20 μm. b) Hoechst and NTP@GOx co-stained images of 4T1 cells collected from 2 to 24 h. Scale bar: 20 μm. c) Relative FL intensity of NTP and NTP@GOx. Images were acquired from high-content screening (PerkinElmer, America) under 660 nm excitation wavelength and 700 nm emission wavelength. Data are means ± SD, *n* = 3.


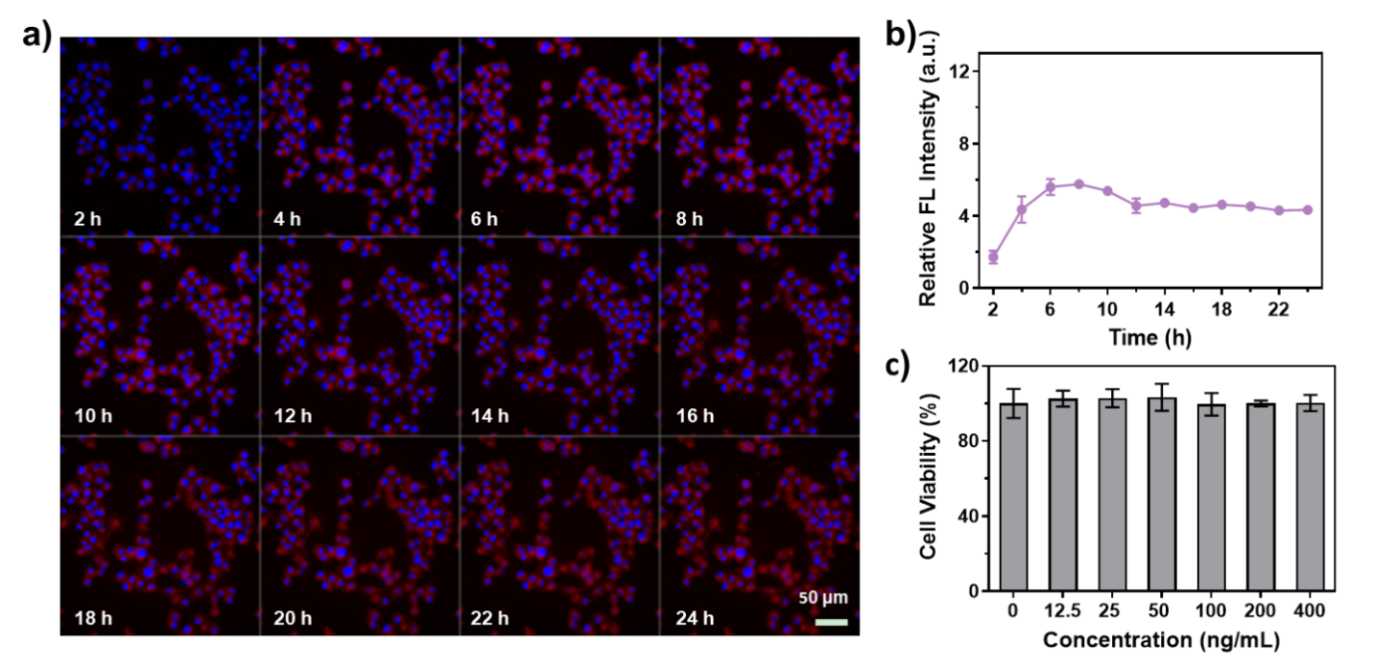


**Figure S24.** Assessment of cellular uptake efficiency and cytotoxicity of NTP@GOx in HEK293T cells. a) Hoechst and NTP@GOx co-stained images and b) relative FL intensity of HEK293T cells collected from 2 to 24 h. Scale bar: 50 μm. Images were acquired from high-content screening under 660 nm excitation and 700 nm emission. c) Relative viabilities of HEK293T cells with NTP@GOx at different concentrations. Data are means ± SD, *n* = 3.


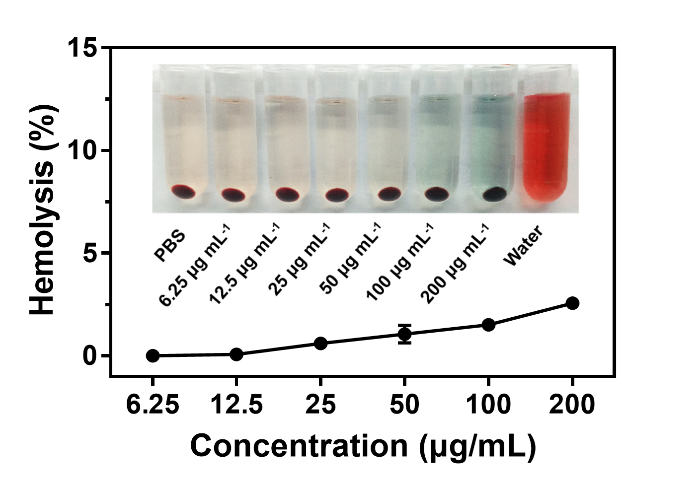


**Figure S25.** Hemolysis of NTP@GOx in different concentrations (6.25-200 µg mL^-1^), while PBS and water were used as positive and negative controls. Data are means ± SD, *n* = 3.


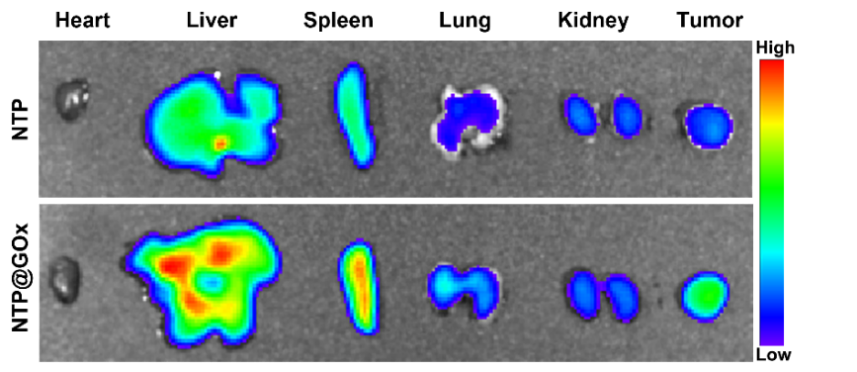


**Figure S26.** *Ex vivo* FL images of tumor tissues and organs of mice after intravenous injection of NTP@GOx or NTP.


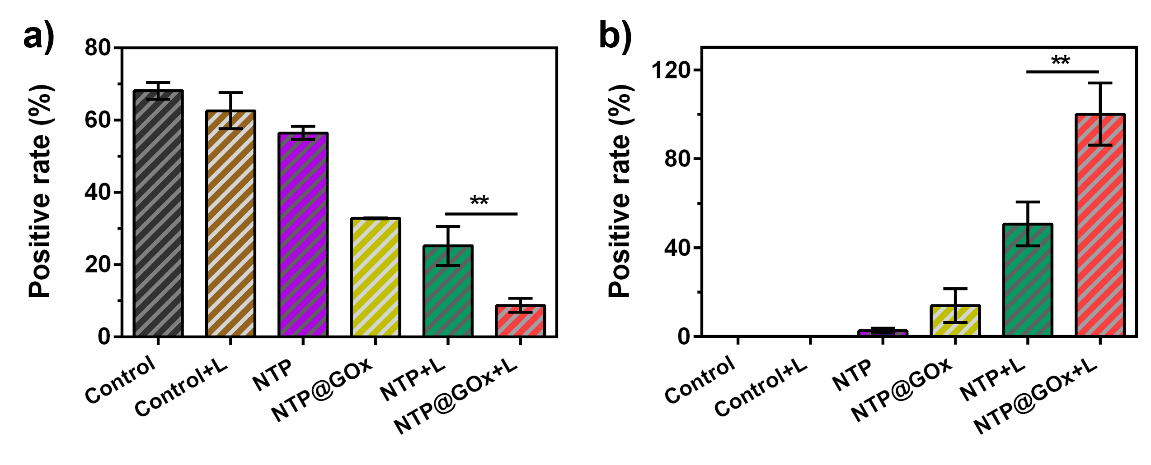


**Figure S27.** Quantification of the positive rates of (a) Ki67 and (b) TUNEL stained images of tumor tissues with indicated treatments. Data are means ± SD, *n* = 3.


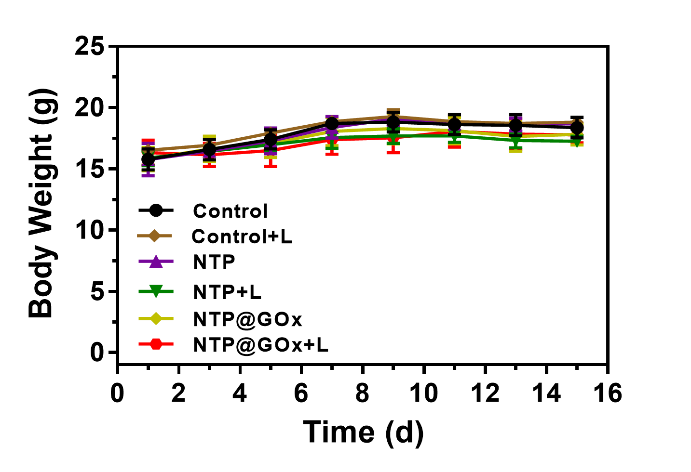


**Figure S28.** Body weight changes of 4T1 tumor-bearing nude mice with various treatments. Data are means ± SD, *n* = 5.


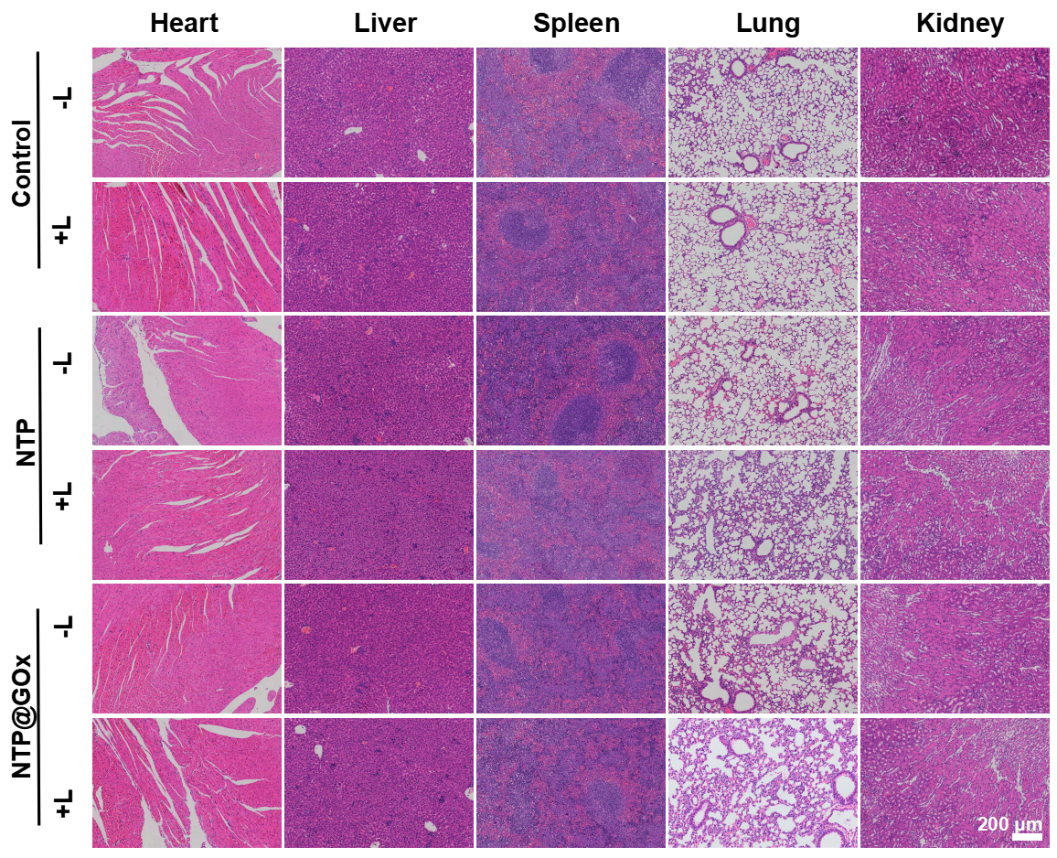


**Figure S29.** H&E-stained images of major organs (heart, liver, spleen, lung, and kidney) of mice with different treatments. Scale bar: 200 μm.


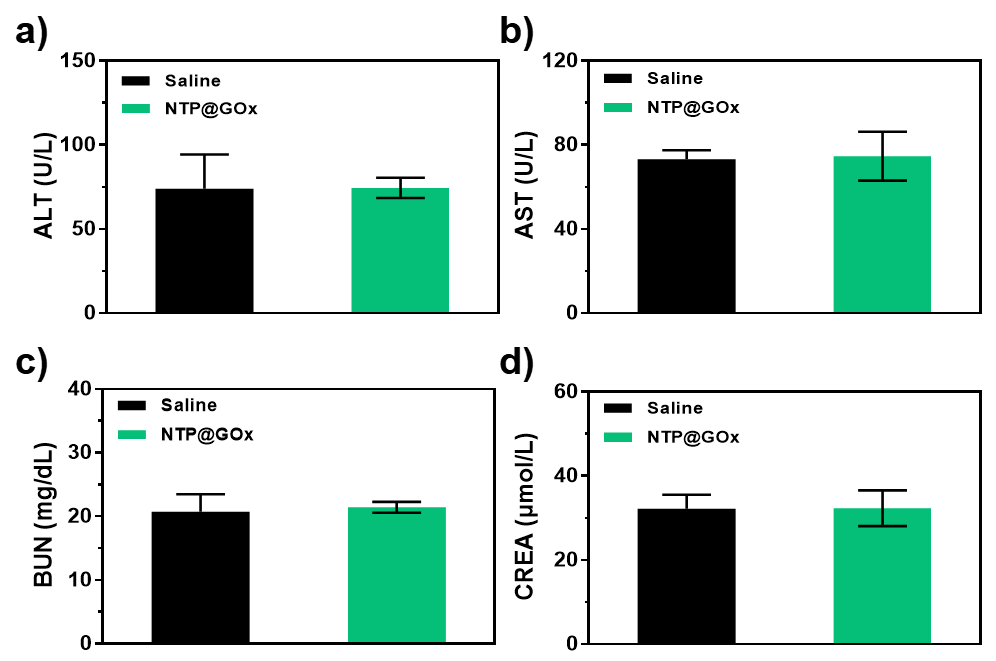


**Figure S30.** Blood biochemistry analysis a) Alanine aminotransferase (ALT), b) aspartate aminotransferase (AST), c) blood urea nitrogen (BUN), and d) creatinine (CREA) of healthy mice with intravenous injection of Saline or NTP@GOx at day 14. Data are means ± SD, *n* = 3.

Table S1. The kinetic parameters of GOx, NTP@GOx, and D-NTP@GOx are obtained by the Michaelis-Menten kinetics plot and the double reciprocal (Lineweaver-Burk) plot.


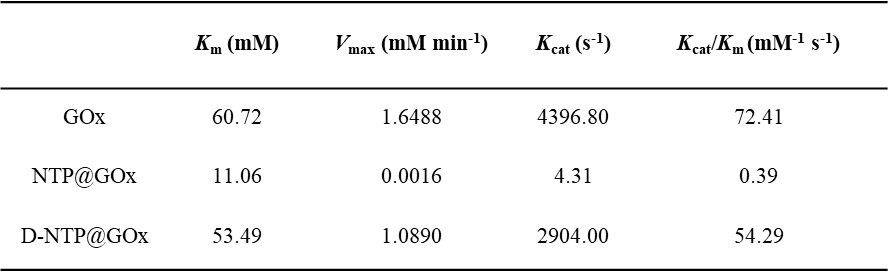


Note: *K*m, Michaelis constant; *V*max, Maximum velocity; *K*cat, Turnover number; *K*cat/*K*m, Catalytic efficiency.

Table S2. The half maximal inhibitory concentration (IC_50_) of 4T1 cells after different treatments calculated by MTT results and graphpad prism software analysis.


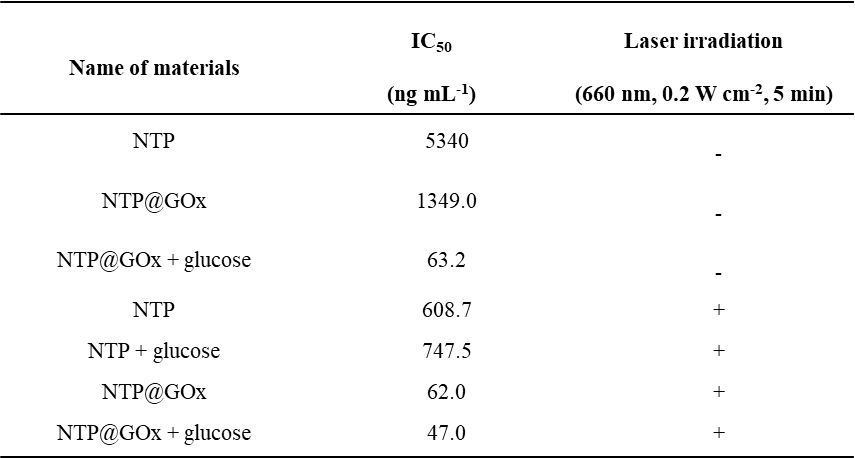

Supplement: Supplementary file 1 — Supporting Information [file ADVS-12-2409960-s001.docx]
